# Glucocorticoid receptor dysregulation underlies 5-HT_2A_R-dependent synaptic and behavioral deficits in a mouse neurodevelopmental disorder model

**DOI:** 10.1016/j.jbc.2022.102481

**Published:** 2022-09-12

**Authors:** Justin M. Saunders, Carolina Muguruza, Salvador Sierra, José L. Moreno, Luis F. Callado, J. Javier Meana, Patrick M. Beardsley, Javier González-Maeso

**Affiliations:** 1 Department of Physiology and Biophysics, Virginia Commonwealth University School of Medicine, Richmond, Virginia, USA; 2 Department of Pharmacology, University of the Basque Country UPV/EHU, CIBERSAM, Biocruces Bizkaia Health Research Institute, Leioa, Bizkaia, Spain; 3 Department of Pharmacology and Toxicology, Virginia Commonwealth University School of Medicine, Richmond, Virginia, USA; 4 Center for Biomarker Research and Precision Medicine, Virginia Commonwealth University School of Pharmacy, Richmond, Virginia, USA

**Keywords:** maternal immune activation, schizophrenia, serotonin 5-HT_2A_ receptor, glucocorticoid receptor, G protein–coupled receptor, neurodevelopmental psychiatric conditions, dendritic spines, AAV, adeno-associated virus, AP, antipsychotic, BSA, bovine serum albumin, cDNA, complementary DNA, ChIP, chromatin immunoprecipitation, CRL, Charles River Laboratories, DEPC, diethyl pyrocarbonate, DMSO, dimethyl sulfoxide, eYFP, enhanced YFP, GR, glucocorticoid receptor, HRP, horseradish peroxidase, 5-HT_2A_R, serotonin 5-HT_2A_ receptor, IL-6, interleukin 6, JAX, Jackson Laboratories, MIA, maternal immune activation, NIH, National Institutes of Health, PFA, paraformaldehyde, poly-(I:C), polyinosinic–polycytidylic acid potassium salt, PPI, prepulse inhibition, qPCR, quantitative PCR, s.c., subcutaneously, SFB, segmented filamentous bacteria, SSC, saline sodium citrate, Tac, Taconic Biosciences, TBST, Tris-buffered saline with Tween-20

## Abstract

Prenatal environmental insults increase the risk of neurodevelopmental psychiatric conditions in the offspring. Structural modifications of dendritic spines are central to brain development and plasticity. Using maternal immune activation (MIA) as a rodent model of prenatal environmental insult, previous results have reported dendritic structural deficits in the frontal cortex. However, very little is known about the molecular mechanism underlying MIA-induced synaptic structural alterations in the offspring. Using prenatal (E12.5) injection with polyinosinic–polycytidylic acid potassium salt as a mouse MIA model, we show here that upregulation of the serotonin 5-HT_2A_ receptor (5-HT_2A_R) is at least in part responsible for some of the effects of prenatal insults on frontal cortex dendritic spine structure and sensorimotor gating processes. Mechanistically, we report that this upregulation of frontal cortex 5-HT_2A_R expression is associated with MIA-induced reduction of nuclear translocation of the glucocorticoid receptor (GR) and, consequently, a decrease in the enrichment of GR at the *5-HT*_*2A*_*R* promoter. The translational significance of these preclinical findings is supported by data in postmortem human brain samples suggesting dysregulation of GR translocation in frontal cortex of schizophrenia subjects. We also found that repeated corticosterone administration augmented frontal cortex *5-HT*_*2A*_*R* expression and reduced GR binding to the *5-HT*_*2A*_*R* promoter. However, virally (adeno-associated virus) mediated augmentation of GR function reduced frontal cortex *5-HT*_*2A*_*R* expression and improved sensorimotor gating processes *via* 5-HT_2A_R. Together, these data support a negative regulatory relationship between GR signaling and *5-HT*_*2A*_*R* expression in the mouse frontal cortex that may carry implications for the pathophysiology underlying *5-HT*_*2A*_*R* dysregulation in neurodevelopmental psychiatric disorders.

Neurodevelopmental psychiatric disorders, such as schizophrenia and autism, are severe and usually cause lifelong disability (1, 2). Epidemiological studies have indicated that maternal infection during pregnancy with a wide variety of agents, including viruses (influenza and rubella) (3, 4, 5, 6, 7), bacteria (bronchopneumonia) (8, 9), and protozoa (*Toxoplasma gondii*) (10), increase the risk of neurodevelopmental psychiatric conditions in the adult offspring. Maternal adverse life events that occur during pregnancy, such as war (11, 12), famine (13), and death or illness in a first-degree relative (14), have also been associated with an elevated risk of schizophrenia and autism. The current consensus is that the effects of prenatal insults are likely explained by their capacity to affect the maternal immune system (15, 16). Accordingly, converging lines of evidence from humans (5) as well as rodent (17) and more recently nonhuman primate (18) animal models suggest a causal relationship between maternal immune activation (MIA) during pregnancy and neuropathological/behavioral abnormalities consistent with a range of neurodevelopmental psychiatric disorders. Previous results based on microarray and RNA-Seq assays have shown changes in gene expression levels that accompany behavioral alterations in rodent MIA models (19, 20, 21). However, there is not presently a clear study assessing the translatability of any of these MIA-affected genes in offspring deficits relevant to neurodevelopmental psychiatric conditions.

The serotonin 5-HT_2A_ receptor (5-HT_2A_R) is a class A G protein–coupled receptor involved in processes related to cognition, perception, and mood (22, 23). Our previous data suggested upregulation of 5-HT_2A_R in postmortem frontal cortex samples of untreated schizophrenia subjects as compared with individually matched controls (24, 25, 26, 27). Interestingly, we also reported a similar phenotype—that is, upregulation of frontal cortex 5-HT_2A_R density—using three independent mouse MIA models that included maternal infection with a mouse-adapted influenza virus (28, 29), maternal variable stress, and MIA with polyinosinic–polycytidylic acid potassium salt (poly-(I:C)) (30). This has been followed by numerous reports revealing upregulation of cortical 5-HT_2A_R in rodent models of environmental insults, such as MIA with poly-(I:C) (31) or lipopolysaccharide (32), maternal stress (33, 34), and lipopolysaccharide administration in adult mice (35), which further strengthens the validity of MIA models to replicate biochemical alterations observed in postmortem schizophrenia frontal cortex samples. It remains unknown, however, whether this 5-HT_2A_R upregulation is involved in the negative effects on disorder-related phenotypes such as dendritic structural plasticity and sensory processes. Furthermore, the upstream signaling mechanisms leading to 5-HT_2A_R upregulation have not been resolved.

Dendritic spines are a fundamental component of synaptic structural plasticity with important roles in cognitive and sensory processes (36). Neuroanatomical studies from postmortem brains of subjects with neurodevelopmental disorders demonstrate altered density and morphology of dendritic spines (37, 38)—particularly in the frontal cortex; a brain region involved in processes related to perception and cognition (39). Our data suggest that MIA-induced upregulation of frontal cortex 5-HT_2A_R occurs through a mechanism that requires dysregulation in nuclear localization and consequently binding of the glucocorticoid receptor (GR) to the promoter region of the *5-HT*_*2A*_*R* gene. In addition, we propose that MIA-induced upregulation of frontal cortex 5-HT_2A_R is at least in part responsible for some of the effects of prenatal insults on frontal cortex dendritic spine structure and sensorimotor gating deficits in the offspring.

## Results

### Poly-(I:C)-induced activation of the immune system in mice

**Figure 1:**
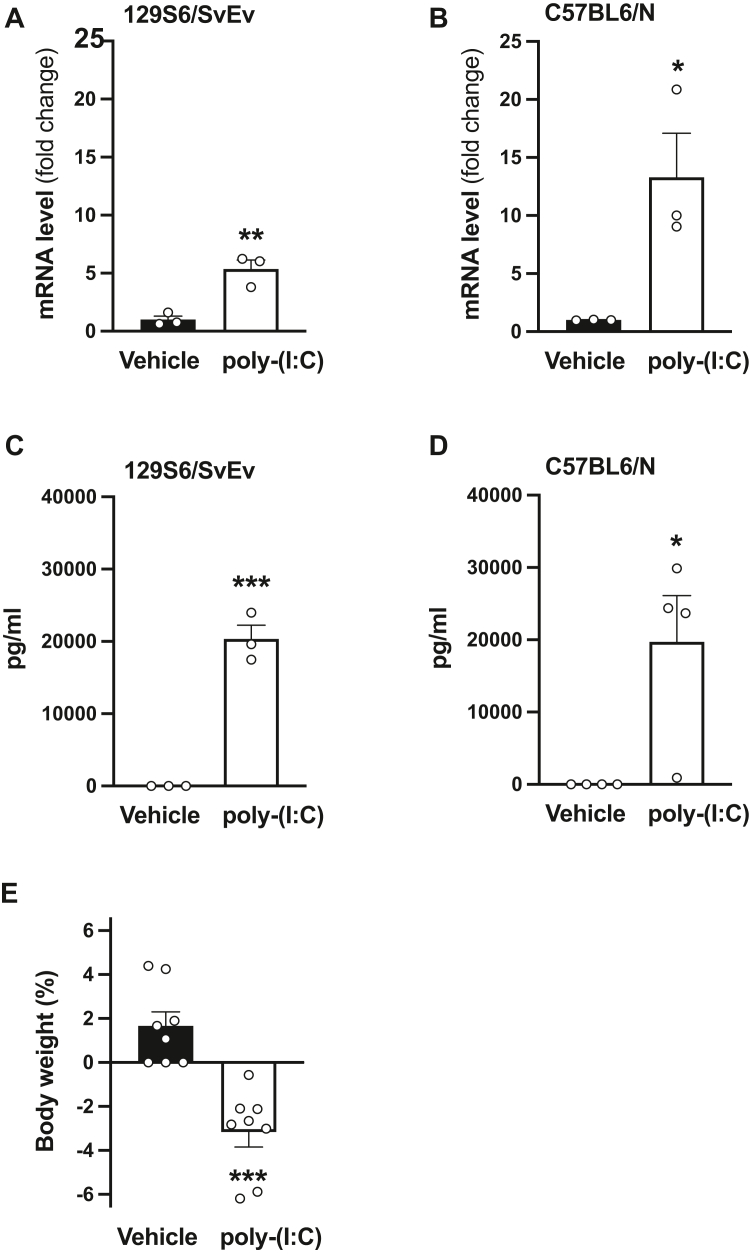
**Poly-(I:C) induces an immune response in nonpregnant female mice**. *A*–*D*, adult female 129S6/SvEv (in-house) (*A* and *C*) and C57BL6/N (CRL) (*B* and *D*) mice were injected (i.p.) with poly-(I:C) (20 mg/kg) or vehicle, and sacrificed 2.5 h after administration. *IL-6* mRNA was assessed by quantitative RT–PCR (qRT–PCR) in mouse frontal cortex samples (*A* and *B*), and IL-6 immunoreactivity was assessed by ELISAs in serum samples (*C* and *D*) (n = 3–4 female mice/group). *E*, adult female C57BL6/N (CRL) mice were injected (i.p.) with poly-(I:C) (20 mg/kg) or vehicle, and percentage change in weight was evaluated 24 h after (n = 8 female mice/group). Data show mean ± SEM. ∗*p* < 0.05, ∗∗*p* < 0.01, and ∗∗∗*p* < 0.001. Unpaired two-tailed Student’s *t* test (*A*: *t*_4_ = 5.21, *p* < 0.01; *B*: *t*_4_ = 3.25, *p* < 0.05; *C*: *t*_4_ = 10.65, *p* < 0.001; *D*: *t*_6_ = 3.07, *p* < 0.05; and *E*: *t*_14_= 5.17, *p* < 0.001). CRL, Charles River Laboratories; poly-(I:C), polyinosinic–polycytidylic acid potassium salt.

Poly-(I:C) is a synthetic analog of dsRNA, a molecular pattern associated with viral infections, that is widely used to provoke an immune response in rodent models, including administration of the viral mimetic to induce immune activation in pregnant mice (17). Recent studies suggest that the immunogenicity and alterations observed in the offspring following administration of poly-(I:C) to pregnant mice may vary in intensity depending on both source and lot of the dsRNA synthetic analogs (40, 41). To assess the effect of poly-(I:C) on the immune response within our experimental system, we tested immunoreactivity and expression of interleukin 6 (IL-6), a key proinflammatory mediator induced by poly-(I:C) administration (42), in serum and frontal cortex samples of nonpregnant adult female 129S6/SvEv (in-house) mice and C57BL6/N mice from Charles River Laboratories (CRL) (see also Table 1 for information regarding inclusion of male and/or female mice throughout). Mice were administered (i.p.) poly-(I:C) (20 mg/kg) or vehicle and sacrificed 2.5 h afterward. Our data show that this model of immune activation leads to an increase in *IL-6* mRNA expression in frontal cortex samples as well as an increase in IL-6 immunoreactivity in serum samples in both 129S6/SvEv (in-house) and C57BL6/N (CRL) animals (Fig. 1, *A–D*). In addition, poly-(I:C)-treated C57BL6/N (CRL) mice showed reduced body weight 24 h after immune activation (Fig. 1*E*).

**Table 1:** Number of litters per group and inclusion of male and/or female mice in the experimental groups

**Figure**	**Number of litters per group**	**Mouse strain**	**Sex**

Figure 1, *A*–*D*	N/A	C57BL6/N and 129S6/SvEv	Female
Figure 1*E*	N/A	C57BL6/N	Female
Figure 2, *A*–*C*	N/A	C57BL6/N, C57BL6/J, 129S6/SvEv	Female
Figure 3, *A* and *B*	2 (Mock) – 3 (MIA)	C57BL6/N	Male
Figure 3, *C* and *D*	3 (Mock) – 4 (MIA)	C57BL6/N	Male and female
Figure 5*A*	N/A	129S6/SvEv	Male
Figure 5, *B*–*F*	7 (Mock) – 10 (MIA)	129S6/SvEv	Male and female
Figure 6*C*	N/A	C57BL6/N	Male
Figure 6, *D* and *E*	3 (Mock) – 4 (MIA)	C57BL6/N	Male
Figure 7, *C* and *D*	3 (Mock) – 4 (MIA)	C57BL6/N	Male
Figure 8, *A*–*C*	N/A	129S6/SvEv	Male
Figure 9, *A*–*G*	N/A	129S6/SvEv	Male
Figure 10*D*	N/A	129S6/SvEv	Male
Figure 11, *A*–*D*	N/A	129S6/SvEv	Male and female
Figure 11, *E*–*H*	N/A	129S6/SvEv	Male
Figure 12, *A*–*D*	N/A	129S6/SvEv	Male
Fig. S1, *B* and *C*	N/A	C57BL6/N	Female

Abbreviation: N/A, not applicable.

**Figure 2:**
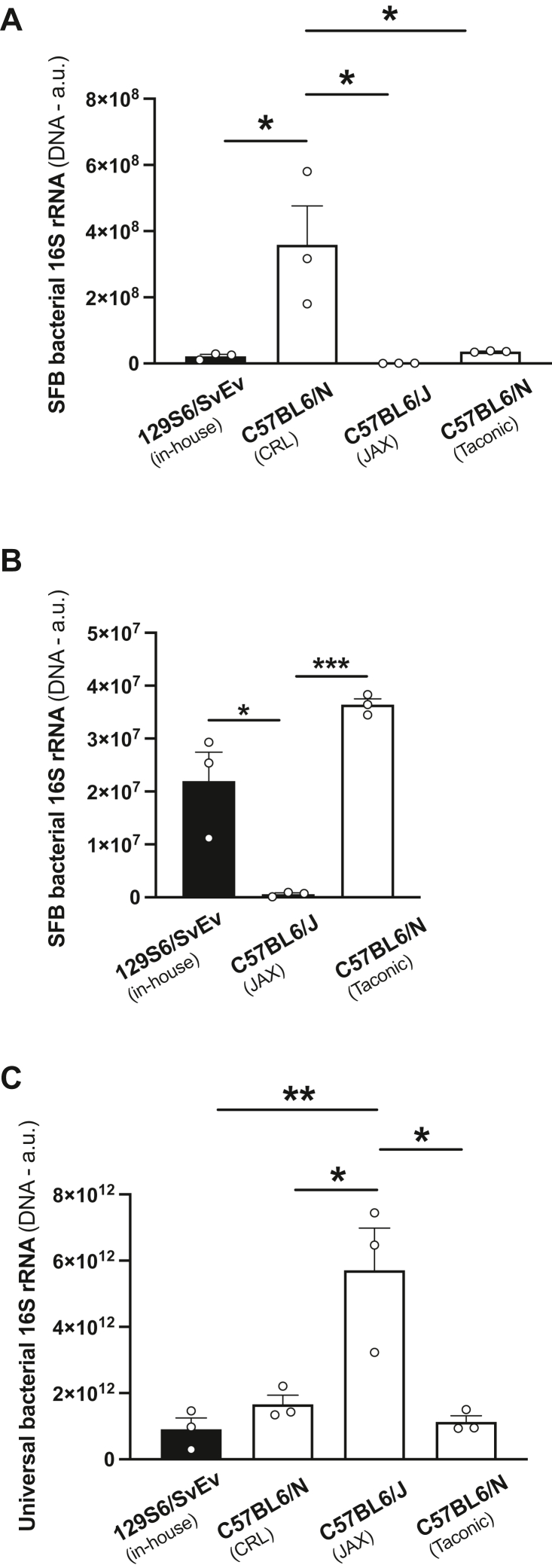
**Gut microbiota are enriched for segmented filamentous bacteria (SFB) in 129S6/SvEv (in-house), C57BL6/N (CRL), and C57BL6/N (Tac), but not C57BL6/J (JAX) adult female mice.***A*–*C*, DNA was extracted from mouse cecum contents, and the 16S rRNA gene was assessed by qPCR using primer pairs targeting either SFB (*A*) or universal primer pairs that detect this gene across the vast majority of bacterial taxa (*C*) (n = 3 female mice/group). *B*, the same data as in “*A*” are depicted, but the C57BL6/N (CRL) mice were excluded from the data analysis. Data show mean ± SEM. ∗*p* < 0.05, ∗∗*p* < 0.01, and ∗∗∗*p* < 0.001. One-way ANOVA followed by Bonferroni’s post hoc test (*A*: *F*[3,8] = 8.40, *p* < 0.01; *B*: *F*[2,6] = 30.95, *p* < 0.001; *C*: *F*[3,8] = 11.9, *p* < 0.01). CRL, Charles River Laboratories; JAX, Jackson Laboratories; qPCR, quantitative PCR; Tac, Taconic Biosciences.

Previous findings have also suggested that maternal gut bacteria composition represents an additional source of variation in neurodevelopmental abnormalities in poly-(I:C)-induced MIA models (43). Particularly, it has been reported that MIA-induced behavioral alterations were more evident in C57BL6/N mice from Taconic Biosciences (Tac) as compared with C57BL6/J from Jackson Laboratories (JAX), and these MIA-induced deficits correlated with the presence of segmented filamentous bacteria (SFB) in the pregnant mother. To evaluate the presence of SFB in female mice, we extracted DNA from cecum contents of 129S6/SvEv (in-house), C57BL6/N (CRL), C57BL6/J (JAX), and C57BL6/N (Tac) animals and performed quantitative PCR (qPCR) assays targeting the *16S rRNA* gene of SFB. Consistent with previous findings (43), our data show that C57BL6/J (JAX) animals were SFB deficient, whereas the presence of SFB was more evident in C57BL6/N (CRL) mice as compared with 129S6/SvEv (in-house) and C57BL6/N (Tac) females (Fig. 2, *A* and *B*). Evaluating the total abundance of the bacterial *16S rRNA* gene using universal qPCR primers that detect this gene across the vast majority of bacterial taxa, we found that C57BL6/N (JAX) mice exhibited a significantly greater abundance of *16S rRNA* gene copies in the cecum as compared with mice from any of the other three sources (Fig. 2*C*).

### MIA-induced upregulation of 5-HT_2A_R and deficits in sensorimotor gating

**Figure 3:**
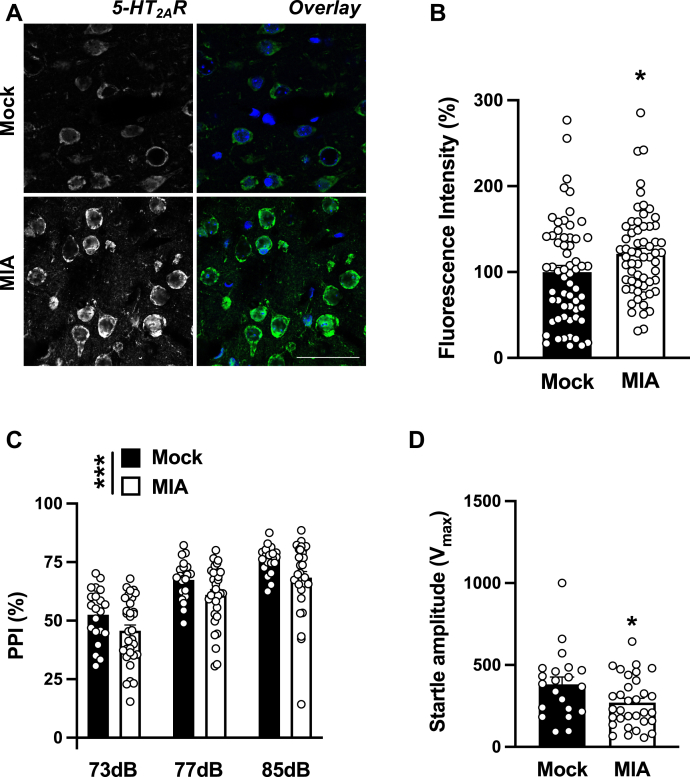
**MIA increases frontal cortex *5-HT***_***2A***_***R* mRNA expression and disrupts sensorimotor gating in the adult offspring**. *A* and *B*, increased expression of *5-HT*_*2A*_*R* mRNA in the frontal cortex of adult MIA mice. Representative FISH images with the antisense *5-HT*_*2A*_*R* probe (*A*), and quantification of fluorescence intensity (n = 61 cells from four male C57BL6/N CRL mice/group; 2–3 litters/group) (*B*). *C* and *D*, effect of MIA on %PPI (*C*) and startle amplitude (*D*) in the adult offspring (n = 21–32 male and female C57BL6/N CRL mice/group; 3–4 litters/group). Data show mean ± SEM. ∗*p* < 0.05, ∗∗∗*p* < 0.001. Unpaired two-tailed Student’s *t* test (*B*: *t*_120_ = 2.12, *p* < 0.05; *D*: *t*_51_ = 2.25, *p* < 0.05). Two-way ANOVA followed by Bonferroni’s post hoc test (*C*: MIA *versus* mock *F*[1,153] = 12.52, *p* < 0.001; prepulse intensity *F*[2,153] = 46.90, *p* < 0.001; interaction *F*[2,153] = 0.04, *p* > 0.05). Nuclei were stained in *blue* with Hoechst (*A*). The scale bar represents 200 μm (*A*). CRL, Charles River Laboratories; *5-HT*_*2A*_*R*, serotonin 5-HT_2A_ receptor; MIA, maternal immune activation; PPI, prepulse inhibition.

Our previous work suggested upregulation of both 5-HT_2A_R density by radioligand-binding assays and *5-HT*_*2A*_*R* mRNA expression by qRT–PCR assays in the frontal cortex of adult mice born to mothers infected with a mouse-adapted influenza virus during pregnancy (28, 29). Using a FISH approach, our data here show that frontal cortex tissue sections from adult MIA offspring exhibit an increase in *5-HT*_*2A*_*R* mRNA relative to mock C57BL6/N offspring mice (Fig. 3, *A* and *B*). Controls to validate the specificity and selectivity of FISH assay included decreased fluorescent signal upon dilution of the mRNA probe (Fig. S1, *A* and *B*) as well as the absence of fluorescent signal in frontal cortex tissue sections exposed to a sense strand *5-HT*_*2A*_*R* mRNA probe (Fig. S1*C*).

Prepulse inhibition (PPI) of startle is a conserved sensorimotor gating behavioral model diminished in individuals with certain psychiatric conditions (44). Similar to previous findings (45), we observed PPI deficits in MIA adult offspring relative to C57BL6/N wildtype controls (Fig. 3*C*). In addition, adult MIA mice exhibited a decrease in startle amplitude during the initial five trials of the PPI paradigm (Fig. 3*D*). A three-way ANOVA demonstrated no sex-related effect on %PPI (Table S1). Similarly, a two-way ANOVA demonstrated no sex-related effect on startle amplitude (MIA: *F*[1,49] = 5.13, *p* < 0.05; sex: *F*[1,49] = 0.04, *p* > 0.05).

Together with our results in Figures 1 and 2, these data corroborate that 129S6/SvEv (in-house) and C57BL6/N (CRL) mice can serve as a preclinical model to evaluate the effects of MIA on neurodevelopmental processes in the offspring.

### MIA-induced reduction of mature dendritic spines *via* 5-HT_2A_R

**Figure 4:**
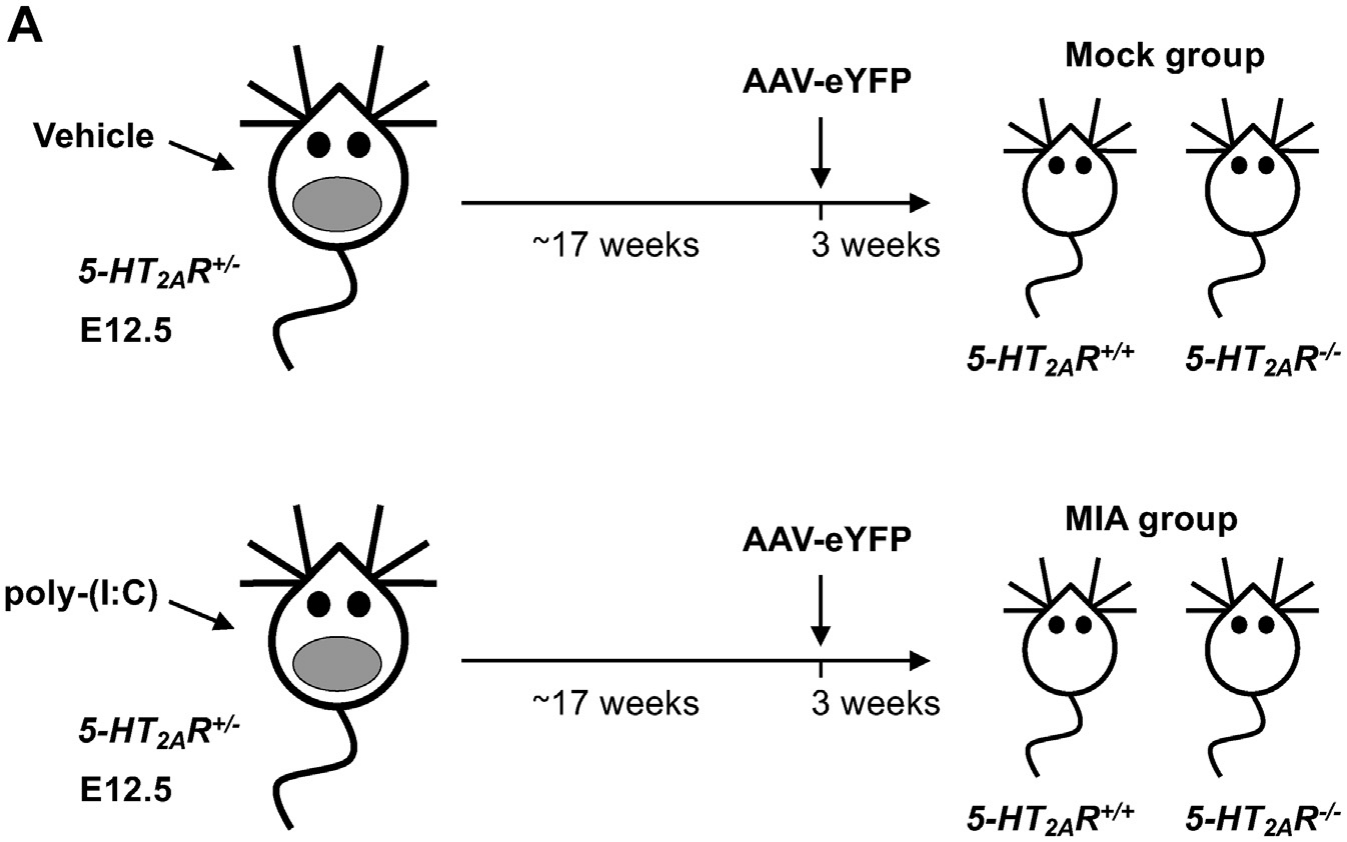
**Breeding *5-HT***_***2A***_***R***^***−/−***^**strategy in MIA mice.***A*, Heterozygous *5-HT*_*2A*_*R*^*+/−*^ mice were bred to obtain *5-HT*_*2A*_*R*^*+/+*^ and *5-HT*_*2A*_*R*^*−/−*^ offspring. Pregnant females (E12.5) received a single injection of poly-(I:C) (20 mg/kg) or vehicle. Adult *5-HT*_*2A*_*R*^*+/+*^ and *5-HT*_*2A*_*R*^*−/−*^ mice born to either mock or MIA mothers were stereotaxically injected with AAV-eYFP and sacrificed for analysis 3 weeks after surgery. AAV, adeno-associated virus; eYFP, enhanced YFP; *5-HT*_*2A*_*R*, serotonin 5-HT_2A_ receptor; MIA, maternal immune activation; poly-(I:C), polyinosinic–polycytidylic acid potassium salt.

**Figure 5:**
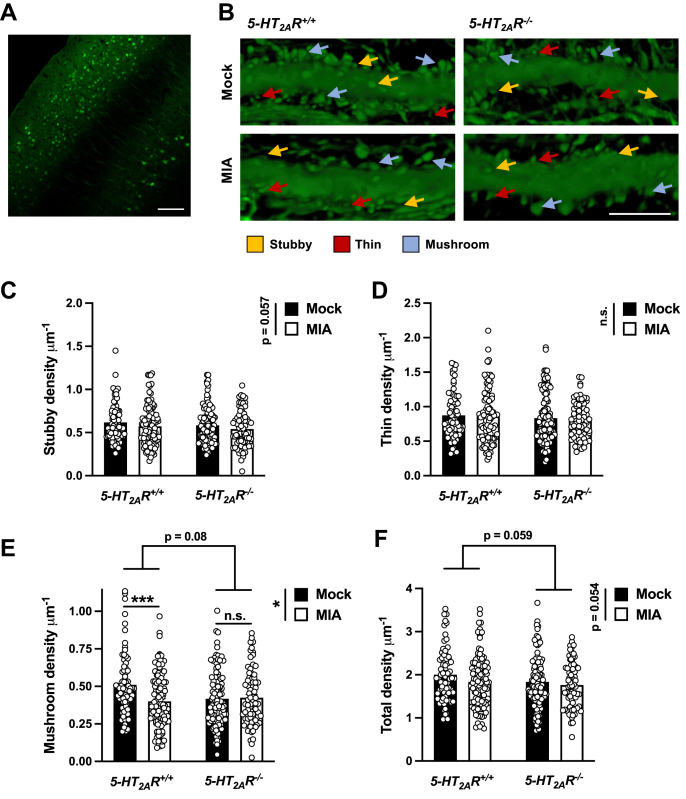
**MIA decreases mature synaptic structural elements in mouse frontal cortex *via* 5-HT**_**2A**_**R.***A*, eYFP expression by immunohistochemistry. *B*, representative three-dimensional reconstructions of AAV-injected cortical dendritic segments. *C*–*F*, stubby (*C*), thin (*D*), mushroom (*E*), and total (*F*) frontal cortex spine density in adult *5-HT*_*2A*_*R*^*+/+*^ and *5-HT*_*2A*_*R*^*−/−*^ mice born to either mock or MIA mothers (n = 73–109 neurons from four to six male and female 129S6/SvEv in-house mice/group; 467-10 litters/group). Data show mean ± SEM. ∗*p* < 0.05, ∗∗∗*p* < 0.001, ns, not significant. Two-way ANOVA followed by Bonferroni’s post hoc test (*C*: stubby MIA *versus* mock *F*[1,363] = 3.64, *p* = 0.057; stubby genotype *F*[1,363] = 1.96, *p* > 0.05; stubby interaction *F*[1,363] = 0.01, *p* > 0.05; *D*: thin MIA *versus* mock *F*[1,363] = 0.48, *p* > 0.05; thin genotype *F*[1,363] = 1.97, *p* > 0.05; thin interaction *F*[1,363] = 0.11, *p* > 0.05; *E*: mushroom MIA *versus* mock *F*[1,363] = 6.58, *p* < 0.05; mushroom genotype *F*[1,363] = 3.01, *p* = 0.08; mushroom interaction *F*[1,363] = 8.45, *p* < 0.05; *F*: total MIA *versus* mock *F*[1,363] = 3.72, *p* = 0.054; total genotype *F*[1,363] = 3.58, *p* = 0.059; total interaction *F*[1,363] = 0.54, *p* > 0.05). The scale bars represent 100 μm (*A*) and 5 μm (*B*). AAV, adeno-associated virus; eYFP, enhanced YFP; 5-HT_2A_R, serotonin 5-HT_2A_ receptor; MIA, maternal immune activation.

Previous findings suggest that MIA affects offspring’s frontal cortex dendritic spine structure (46, 47, 48). To determine whether alterations in frontal cortex 5-HT_2A_R-dependent signaling are involved in the effects of MIA on offspring’s frontal cortex dendritic structural plasticity, heterozygous *5-HT*_*2A*_*R*^*+/−*^ 129S6/SvEv pregnant mice were injected with poly-(I:C) or vehicle, and cortical dendritic structure assays were tested in adult *5-HT*_*2A*_*R*^*+/+*^ or *5-HT*_*2A*_*R*^*−*/*−*^ offspring (Fig. 4*A*). To target cortical pyramidal neurons, adult MIA and mock mice were stereotaxically injected into the frontal cortex with adeno-associated virus serotype 8 (AAV8) preparations expressing enhanced YFP (eYFP) under the control of the *CaMKIIα* promoter (Fig. 5*A*). Using a similar viral vector, our previous findings reported a robust overexpression of eYFP in excitatory pyramidal CaMKIIα-positive but not inhibitory parvalbumin-positive neurons (49). In slices prepared approximately 3 weeks after viral injection, when transgene expression is maximal, this approach enabled us to explicitly discriminate single pyramidal cells and their dendritic spines. Notably, MIA offspring mice showed a selective reduction of mature mushroom spines in CaMKIIα-positive neurons (Fig. 5, *B* and *E*). There was also a trend toward MIA effect on stubby spines (Fig. 5, *B* and *C*) and total spine density (Fig. 5, *B* and *F*), whereas immature thin spines remained unaffected (Fig. 5, *B* and *D*). This effect of MIA required expression of 5-HT_2A_R since the frontal cortex synaptic remodeling alteration was not observed in *5-HT*_*2A*_*R*^*−/−*^ littermates (Fig. 5, *B–F*). Similar to previous reports (50), adult mice with selective deletion of the *5-HT*_*2A*_*R* gene presented a trend toward reduction in the density of frontal cortex dendritic spines (Fig. 5, *B–F*). A three-way ANOVA demonstrated no sex-related effect on MIA-induced alterations in the density of mature mushroom spines. However, a three-way ANOVA suggested sex-related differences in immature thin spines and total spine density (Tables S2–S5).

### Reduced GR binding to the 5-HT_2A_R promoter in MIA mice

**Figure 6:**
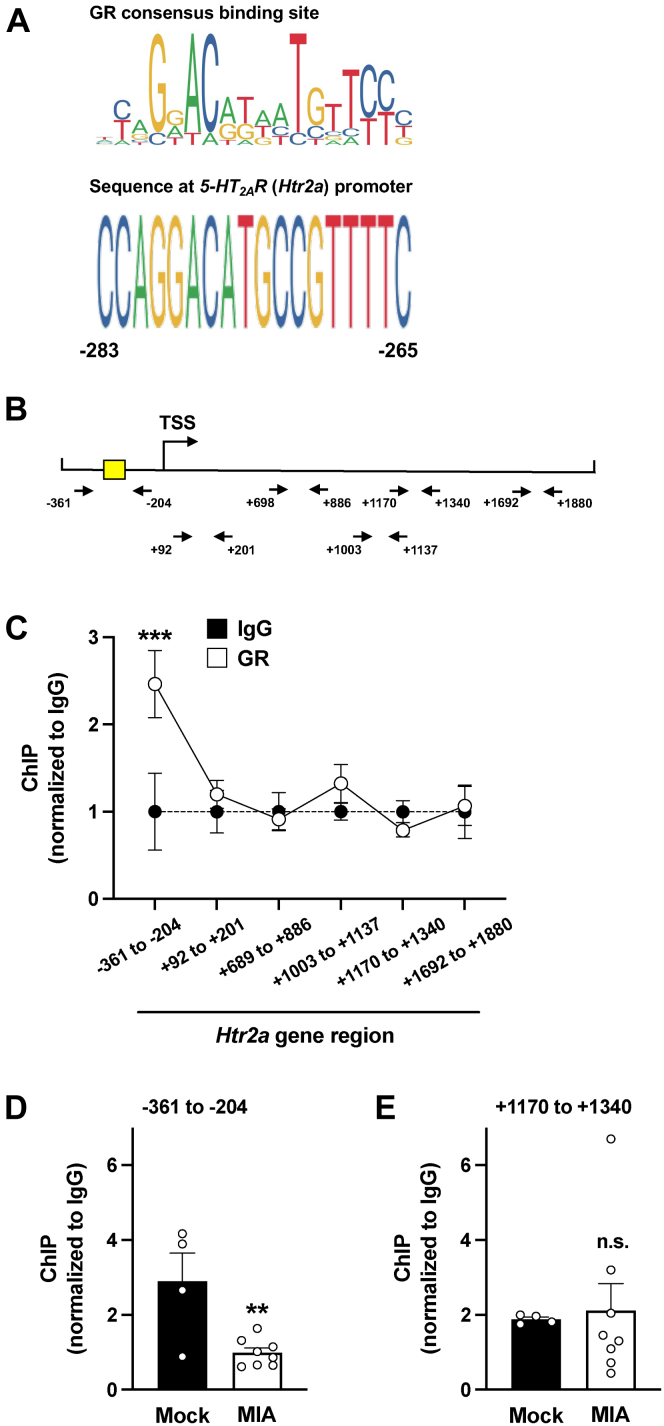
**MIA dysregulates binding of GR to the *5-HT***_***2A***_***R* promoter in mouse frontal cortex**. *A*, alignment of the vertebrate GR consensus binding site with the promoter of the mouse *5-HT*_*2A*_*R* gene. *B*, map of the *5-HT*_*2A*_*R* gene showing position of primers used for qPCR assays. *Yellow box* indicates location of the predicted GR-binding site. *C*, GR binds to the promoter region of the *5-HT*_*2A*_*R* gene in mouse frontal cortex (n = 8 male C57BL6/N CRL mice/group). *D* and *E*, binding of GR to the region of the *5-HT*_*2A*_*R* gene containing a putative GR-binding site (*D*), but not within exon 1 (*E*), is decreased in frontal cortex samples of adult MIA mice (n = 4–8 male C57BL6/N CRL mice/group; 3–4 litters/group). Data show mean ± SEM. ∗∗*p* < 0.01, ∗∗∗*p* < 0.001, ns, not significant. Two-way ANOVA followed by Bonferroni’s post hoc test (*C*: antibody *F*[1,73] = 3.83, *p* = 0.054; gene site *F*[5,73] = 2.80, *p* < 0.05). Unpaired two-tailed Student’s *t* test (*D*: *t*_10_ = 3.57, *p* < 0.01; *E*: *t*_10_ = 0.23, *p* > 0.05). CRL, Charles River Laboratories; GR, glucocorticoid receptor; *5-HT*_*2A*_*R*, serotonin 5-HT_2A_ receptor; MIA, maternal immune activation; qPCR, quantitative PCR; TSS, transcriptional start site.

GRs, which belong to an evolutionary conserved nuclear receptor superfamily, are involved in the mechanism by which steroid hormones, such as cortisol in most mammals including humans and corticosterone in laboratory rodents, regulate stress responsiveness as ligand-dependent transcription factors across different species (51). Previous reports raise the possibility that the GR regulates *5-HT*_*2A*_*R* transcription *in vitro* in different experimental systems in a manner expected to be conserved from mice to humans (52), but the basic molecular mechanism underlying crosstalk between the GR and serotonin receptor systems *in vivo* remains unsolved. Using the consensus sequence for the GR obtained from transcription factor–binding datasets (53), we detected a predicted GR-binding site at the promoter region of the mouse *5-HT*_*2A*_*R* gene (Fig. 6*A*). Based on these findings, we performed a series of chromatin immunoprecipitation followed by qPCR (ChIP–qPCR) assays with an anti-GR antibody in mouse frontal cortex samples throughout different regions of the *5-HT*_*2A*_*R* gene (Fig. 6*B*). Our ChIP–qPCR data revealed enrichment of GR at the predicted binding site but not at other locations along the *5-HT*_*2A*_*R* gene (Fig. 6*C*).

Following this finding in naïve mice, we next sought to determine the effect of MIA on GR enrichment at the *5-HT*_*2A*_*R* promoter in mouse frontal cortex. Notably, ChIP–qPCR data reveal that prenatal MIA leads to a significant decrease in the enrichment of GR at the *5-HT*_*2A*_*R* promoter (Fig. 6*D*) but not at a different location within exon 1 (Fig. 6*E*).

### Dysregulated GR translocation in MIA mice and schizophrenia brain samples

**Figure 7:**
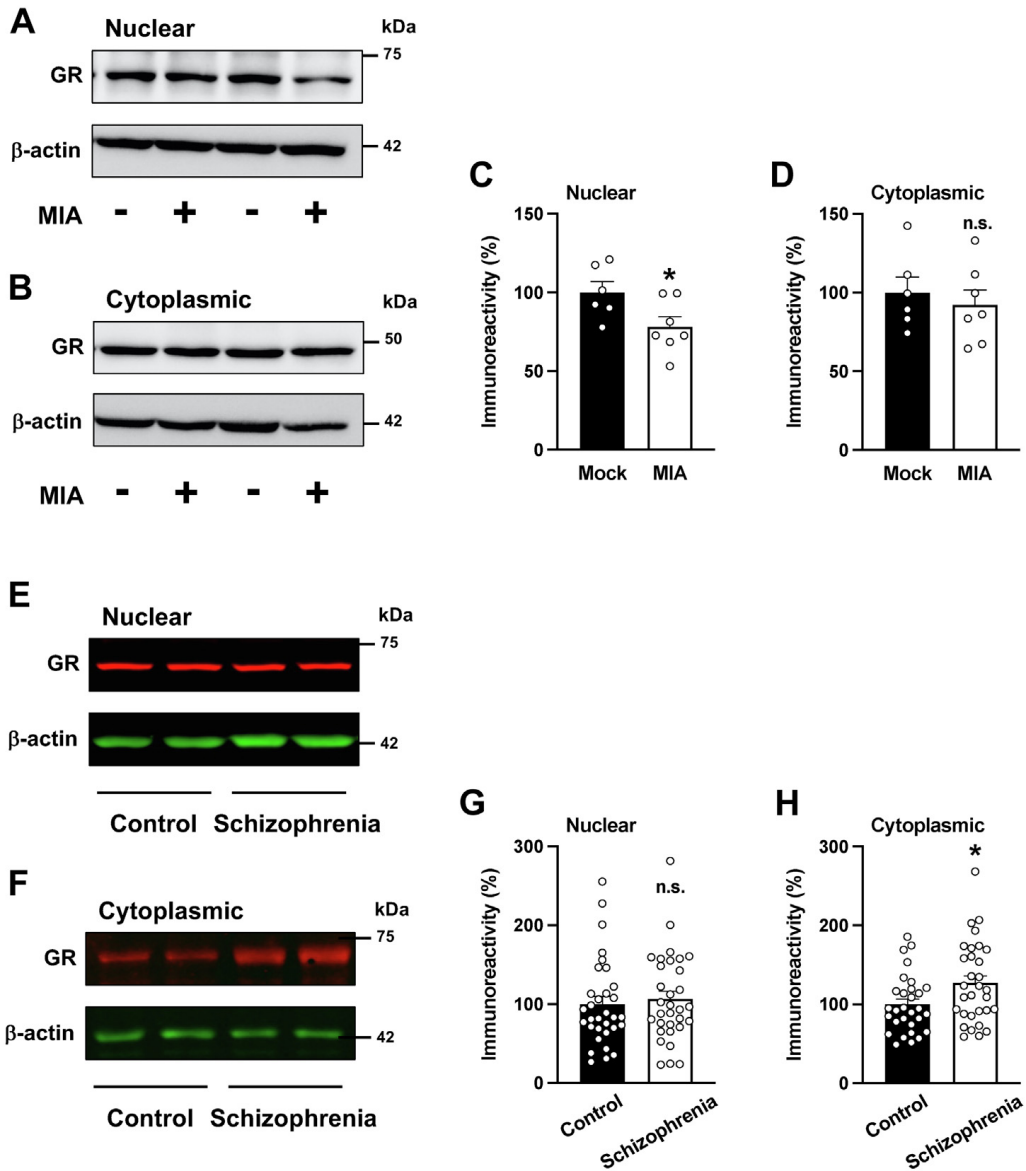
**Dysregulation of GR localization in MIA mice and postmortem frontal cortex samples of schizophrenia subjects.***A*–*D*, immunoblots showed downregulation of GR protein in nuclear (*C*) but not cytoplasmic (*D*) preparations from the frontal cortex of adult MIA mice as compared with mock animals. Representative immunoblots are shown (*A* and *B*) (n = 6–7 male C57BL6/N CRL mice/group; 3–4 litters/group). *E*–*H*, immunoblots showed upregulation of GR protein in cytoplasmic (*H*) but not nuclear (*G*) preparations from postmortem human brain samples of schizophrenia subjects as compared with controls (for demographic information, see Tables 2 and 3). Representative immunoblots are shown (*E* and *F*). Data show mean ± SEM. ∗*p* < 0.05, ns, not significant. Unpaired two-tailed Student’s *t* test (*C*: *t*_11_ = 2.34, *p* < 0.05; *D*: *t*_11_ = 0.57, *p* > 0.05; *G*: *t*_62_ = 0.47, *p* > 0.05; *H*: *t*_59_ = 2.35, *p* < 0.05). GR, glucocorticoid receptor; MIA, maternal immune activation.

To gain insights into a potential mechanism underlying these findings, and given that activation of the GR pathway can induce GR nuclear translocation, we tested whether prenatal MIA affects subcellular localization of GR in mouse frontal cortex. Consistent with previous studies, GR immunoreactivity was observed in both nuclear and cytoplasmic preparations of frontal cortex samples from control mice (Fig. 7, *A* and *B*). Importantly, MIA offspring animals showed decreased immunoreactivity against nuclear (Fig. 7, *A* and *C*), but not cytoplasmic (Fig. 7, *B* and *D*), GR in the frontal cortex.

To test for potential dysregulation of GR trafficking in individuals with neurodevelopmental psychiatric conditions, we performed cellular fractionation followed by immunoblot assays with anti-GR antibodies in postmortem frontal cortex samples of schizophrenia subjects and individually matched controls (Tables 2 and 3). Interestingly, we observed a statistically significant difference related to GR subcellular localization in frontal cortex samples from schizophrenia subjects as compared with controls (Fig. 7, *E–H*). This alteration in GR nuclear translocation was also apparent as a trend in both antipsychotic-free and antipsychotic-treated schizophrenia subjects *versus* their respective control groups (nuclear: antipsychotic-free *versus* controls, Student’s *t* test, *t*_30_ = 1.09, *p* > 0.05; antipsychotic-treated *versus* controls, Student’s *t* test, *t*_30_ = 0.45, *p* > 0.05) (cytoplasmic: antipsychotic-free *versus* controls, Student’s *t* test, *t*_28_ = 1.49, *p* = 0.14; antipsychotic-treated *versus* controls, Student’s *t* test, *t*_29_ = 1.85, *p* = 0.07).

**Table 2:** Demographic characteristics of antipsychotic-free schizophrenia subjects and their respective control subjects

	Sex (F/M)	Age at death (years)	Postmortem delay (h)	Storage time (months)	Antipsychotic treatment	Additional drugs
Schizophrenia 1	M	30	51	203	(−)	(−)
Control 1	M	29	18	68		(−)
Schizophrenia 2	M	48	20	122	(−)	(−)
Control 2	M	47	18	142		BDZ
Schizophrenia 3	M	31	11	113	(−)	(−)
Control 3	M	31	13	99		ETH (0.96 g/l)
Schizophrenia 4	M	45	3	106	(−)	BDZ
Control 4	M	44	21	92		(−)
Schizophrenia 5	M	27	24	104	(−)	(−)
Control 5	M	28	30	178		(−)
Schizophrenia 6	M	46	22	69	(−)	(−)
Control 6	M	46	24	67		(−)
Schizophrenia 7	M	48	11	50	(−)	(−)
Control 7	M	49	8	43		(−)
Schizophrenia 8	M	45	18	59	(−)	(−)
Control 8	M	47	15	44		(−)
Schizophrenia 9	M	34	15	62	(−)	(−)
Control 9	M	36	48	117		(−)
Schizophrenia 10	M	52	7	66	(−)	BDZ
Control 10	M	51	13	46		(−)
Schizophrenia 11	M	49	12	33	(−)	BDZ
Control 11	M	46	6	28		ETH (1.91 g/l)
Schizophrenia 12	M	60	12	43	(−)	BDZ
Control 12	M	62	9	66		(−)
Schizophrenia 13	F	67	22	52	(−)	(−)
Control 13	F	66	16	24		(-)
Schizophrenia 14	M	34	23	116	(−)	(−)
Control 14	M	33	28	28		ETH (0.97 g/l)
Schizophrenia 15	M	32	52	121	(−)	BDZ
Control 15	M	32	4	44		ETH (2.98 g/l)
Schizophrenia 16	M	32	21	122	(−)	(−)
Control 16	M	32	16	33		(−)
Schizophrenia	1F/15M	42.5 ± 2.9	20.3 ± 3.4	90.1 ± 10.9		
Control	1F/15M	42.4 ± 2.9	17.9 ± 2.7	69.9 ± 11.2		

Abbreviations: BDZ, benzodiazepine; ETH, ethanol; F, female; M, male.

Antipsychotics were not detected in blood samples of schizophrenia subjects at the time of death.

**Table 3:** Demographic characteristics of antipsychotic-treated schizophrenia subjects and their respective control subjects

	Sex (F/M)	Age at death (years)	Postmortem delay (h)	Storage time (months)	Antipsychotic treatment	Additional drugs
Schizophrenia 17	M	30	18	130	OLZ	(−)
Control 17	M	29	36	185		ETH (1.71 g/l)
Schizophrenia 18	M	32	8	125	QTP	BDZ
Control 18	M	32	4	24		(−)
Schizophrenia 19	M	23	16	120	SUL	(−)
Control 19	M	23	17	93		(−)
Schizophrenia 20	M	35	3	120	QTP	BDZ
Control 20	M	36	23	63		(−)
Schizophrenia 21	M	33	23	107	CLZ	(−)
Control 21	M	33	4	99		(−)
Schizophrenia 22	M	35	11	70	CLZ	BDZ
Control 22	M	36	18	140		ETH (1.69 g/l)
Schizophrenia 23	F	42	38	129	CLZ, QTP, SUL	BDZ
Control 23	F	38	22	46		(−)
Schizophrenia 24	M	37	8	75	OLZ	BDZ, ETH (0.90 g/l)
Control 24	M	41	24	180		(−)
Schizophrenia 25	F	60	23	54	CLZ, SUL	BDZ
Control 25	F	60	48	60		BDZ
Schizophrenia 26	F	56	13	119	CLZ	(−)
Control 26	F	58	20	176		(−)
Schizophrenia 27	M	43	5	21	OLZ	BDZ
Control 27	M	44	29	24		(−)
Schizophrenia 28	M	58	6	21	CLP	(−)
Control 28	M	57	3	39		(−)
Schizophrenia 29	M	51	18	22	PAL	BDZ
Control 29	M	50	2	33		(−)
Schizophrenia 30	M	51	28	25	OLZ,CLP	BDZ
Control 30	M	50	24	20		(−)
Schizophrenia 31	M	34	21	28	HAL	BDZ
Control 31	M	33	17	33		(−)
Schizophrenia 32	M	58	16	33	APZ	(−)
Control 32	M	56	15	18		(−)
Schizophrenia	3F/13M	42.4 ± 2.9	15.9 ± 2.3	74.9 ± 11.4		
Control	3F/13M	42.3 ± 2.9	19.1 ± 3.1	77.1 ± 15.3		

Abbreviations: BDZ, benzodiazepine; ETH, ethanol.

Therapeutic levels of aripriprazole (APZ), clotiapine (CLP), clozapine (CLZ), haloperidol (HAL), olanzapine (OLZ), paliperidone (PAL), quetiapine (QTP), and sulpiride (SUL) were detected in blood samples of schizophrenia subjects at the time of death.

### Repeated corticosterone administration upregulates 5-HT_2A_R expression in mice

**Figure 8:**
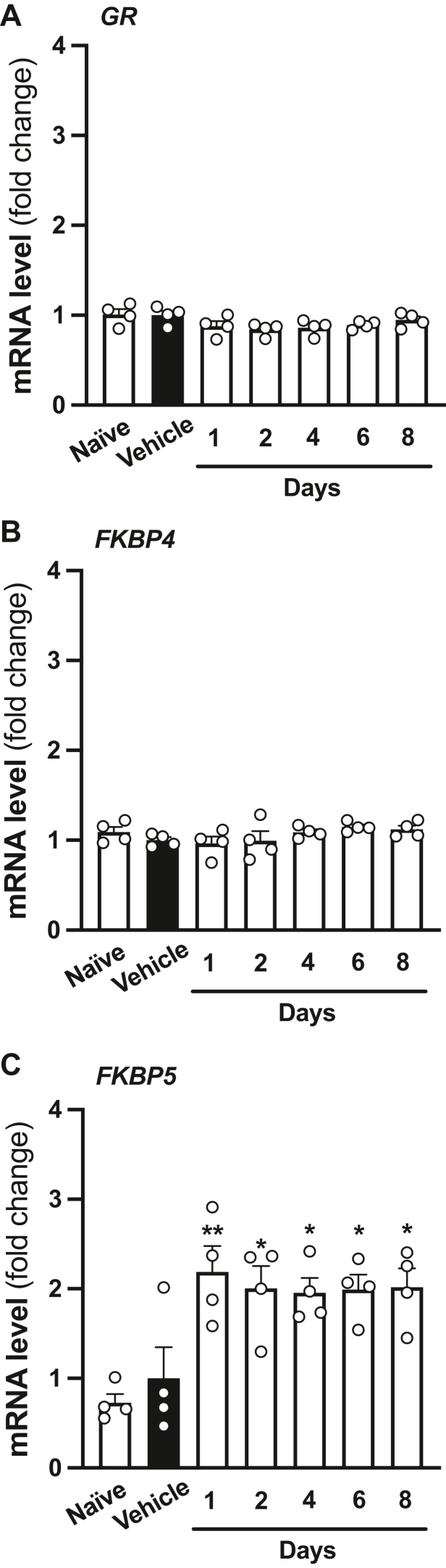
**A time course of repeated corticosterone administration reveals *FKBP5* mRNA induction in mice.***A*–*C*, adult male mice were administered (s.c.) corticosterone (50 mg/kg) twice a day for either 1, 2, 4, 6, or 8 days and sacrificed to collect samples 8.5 to 13 h after the last injection. Control groups included mice injected with vehicle as well as injection-naïve mice. Expression of *GR* (*A*), *FKBP4* (*B*), and *FKBP5* (*C*) mRNAs in frontal cortex was assessed by qRT–PCR (n = 4 male 129S6/SvEv in-house mice/group). Data show mean ± SEM. ∗*p* < 0.05, ∗∗*p* < 0.01. One-way ANOVA followed by Dunnett’s post hoc test (*A*: *F*[6,21] = 2.17, *p* > 0.05; *B*: *F*[6,21] = 1.38, *p* > 0.05; *C*: *F*[6.21] = 6.26, *p* < 0.001). s.c., subcutaneously; qRT, quantitative RT.

Following our data suggesting that MIA produces both upregulation of *5-HT*_*2A*_*R* mRNA expression and decreased GR binding to the *5-HT*_*2A*_*R* promoter in mouse frontal cortex, we focused our investigation on the effects of direct manipulation of the GR system on 5-HT_2A_R-related behavioral phenotypes. Previous investigation has reported that a regimen of corticosterone twice a day for 4 days produces an increase in head-twitch behavior—a rodent behavioral proxy of human hallucinogenic potential (54)—in response to the psychedelic 5-HT_2A_R agonist 1-(2,5-dimethoxy-4-iodophenyl)-2-aminopropane in rats (55). To evaluate and optimize this experimental system in 129S6/SvEv mice, we first administered (subcutaneously [s.c.]) corticosterone (50 mg/kg) twice a day for up to 8 days and sacrificed 8.5 to 13 h after the last injection. Control groups included mice injected with vehicle as well as injection-naïve mice. Previous findings have established FKBP5 as an interactor that opposes nuclear translocation of the GR and hence decreases GR-dependent transcriptional activity. Upon glucocorticoid binding, however, FKBP5 is exchanged for FKBP4, a cochaperone that promotes GR nuclear translocation and transcriptional regulation (56). Our data suggest that neither *GR* (Fig. 8*A*) nor *FKBP4* (Fig. 8*B*) mRNA expression was affected upon repeated corticosterone administration. However, *FKBP5* mRNA (Fig. 8*C*) showed upregulation in the frontal cortex of mice after either 1, 2, 4, 6, or 8 days of repeated corticosterone administration, as compared with vehicle; the injection-naïve group was not significantly different from vehicle. These findings suggest that repeated corticosterone administration activates and elicits negative feedback within the GR pathway in the mouse frontal cortex. We therefore elected to use 4 days as the treatment duration for our studies.

**Figure 9:**
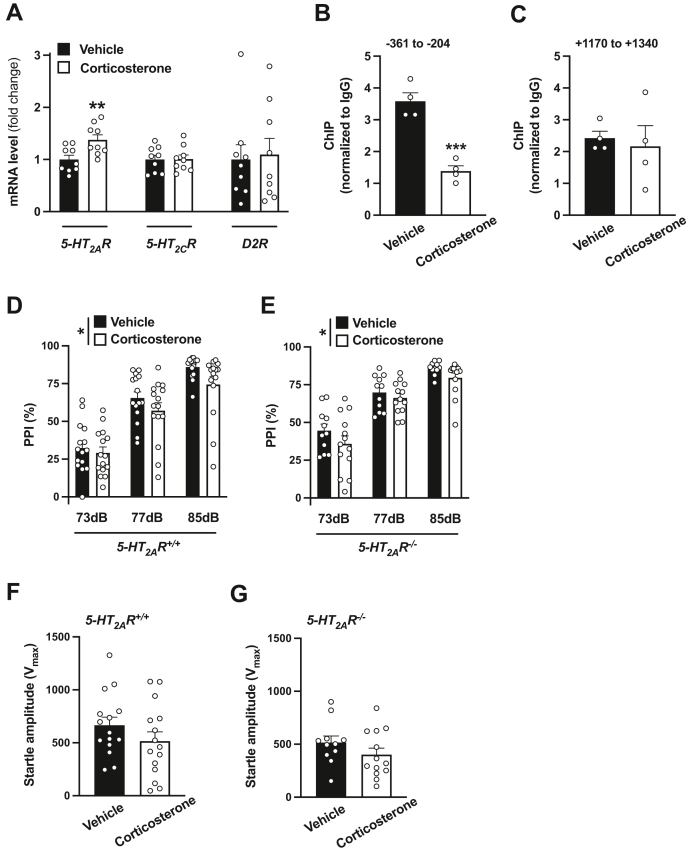
**Repeated corticosterone administration upregulates frontal cortex *5-HT***_***2A***_***R* mRNA expression and disrupts sensorimotor gating in mice.***A*, increased expression of *5-HT*_*2A*_*R* mRNA in frontal cortex after repeated corticosterone administration. Mice were treated (twice a day for 4 days) with corticosterone (50 mg/kg; s.c.) or vehicle and sacrificed for analysis 8.5 to 13.5 h after the last injection. Expression of *5-HT*_*2A*_*R*, *5-HT*_*2C*_*R*, and *D*_*2*_*R* mRNAs in frontal cortex was assessed by qRT–PCR (n = 9 male 129S6/SvEv mice/group). *B* and *C*, enrichment of the GR at the region of the *5-HT*_*2A*_*R* gene containing a putative GR-binding site (*B*), but not within exon 1 (*C*), is decreased upon repeated corticosterone administration in mouse frontal cortex (n = 4 male 129S6/SvEv mice/group). *D*–*G*, effect of repeated corticosterone administration on %PPI (*D* and *E*) and startle amplitude (*F* and *G*) in *5-HT*_*2A*_*R*^*+/+*^ (n = 15 male 129S6/SvEv mice/group) and *5-HT*_*2A*_*R*^*−/−*^ (n = 11–13 male 129S6/SvEv mice/group) animals. Data show mean ± SEM. ∗*p* < 0.05, ∗∗*p* < 0.01, and ∗∗∗*p* < 0.001. Unpaired two-tailed Student’s *t* test (*A*: *5-HT*_*2A*_*R t*_16_ = 2.98, *p* < 0.01; *5-HT*_*2C*_*R t*_16_ = 0.06, *p* > 0.05; *D*_*2*_*R t*_16_ = 0.23, *p* > 0.05; *B*: *t*_6_ = 6.95, *p* < 0.001; *C*: *t*_6_ = 0.38, *p* > 0.05; *F*: *t*_28_ = 1.28, *p* > 0.05; *G*: *t*_22_ = 1.30, *p* > 0.05). Two-way ANOVA followed by Bonferroni’s post hoc test (*B*: corticosterone *versus* vehicle *F*[1,84] = 4.87, *p* < 0.05; prepulse intensity *F*[2,84] = 67.28, *p* < 0.001; interaction *F*[2,84] = 0.47, *p* > 0.05; *E*: corticosterone *versus* vehicle *F*[1,66] = 4.20, *p* < 0.05; prepulse intensity *F*[2,66] = 66.62, *p* < 0.001; interaction *F*[2,66] = 0.24, *p* > 0.05; for three-way ANOVA, see Table 4). *D*_*2*_*R*, dopamine D2 receptor; GR, glucocorticoid receptor; *5-HT*_*2A*_*R*, serotonin 5-HT_2A_ receptor; PPI, prepulse inhibition; qRT, quantitative RT; s.c., subcutaneously.

Upon repeated (twice a day for 4 days) administration (s.c.) of corticosterone (50 mg/kg), we found upregulation of *5-HT*_*2A*_*R* mRNA expression in the mouse frontal cortex, an effect that was not observed with the closely related *5-HT*_*2C*_*R* or the *dopamine D_2_ receptor* (Fig. 9*A*). In addition, this paradigm of repeated corticosterone administration led to a decrease of GR enrichment at its predicted binding site within the *5-HT*_*2A*_*R* promoter (Fig. 9*B*), an effect that was not observed at a region within exon 1 (Fig. 9*C*).

Using the same model of repeated corticosterone administration, we next evaluated the effects on sensorimotor gating and the role of 5-HT_2A_R-dependent signaling in mediating this behavior. Corticosterone treatment reduced %PPI in *5-HT*_*2A*_*R*^*+/+*^ mice (Fig. 9*D*), an effect that was also observed in *5-HT*_*2A*_*R*^*−/−*^ mice (Fig. 9*E*). Startle magnitude was unaffected upon repeated corticosterone treatment (Fig. 9, *F* and *G*), whereas *5-HT*_*2A*_*R*^*−/−*^ mice showed a statistically significant increase in %PPI as a genotype effect compared with *5-HT*_*2A*_*R*^*+/+*^ controls (Fig. 9, *D* and *E*, and Table 4). These data suggest that although repeated corticosterone treatment augments *5-HT*_*2A*_*R* mRNA expression in mouse frontal cortex—an effect that occurs in parallel with decreased binding of the GR the *5-HT*_*2A*_*R* promoter—the negative effect of this repeated pharmacological activation of the GR on sensorimotor gating processes does not seem to be dependent on 5-HT_2A_R-dependent signaling mechanisms.

**Table 4:** Three-way ANOVA of the effect of repeated corticosterone *versus* vehicle treatment on %PPI in male 129S6/SvEv (in-house) mice

Source of variation	ANOVA	*p*
Prepulse intensity	*F*[2,150] = 125.7	*p* < 0.001
Treatment	*F*[1,150] = 8.55	*p* < 0.01
Genotype	*F*[1,150] = 6.84	*p* < 0.01
Prepulse intensity × treatment	*F*[2,150] = 0.17	*p* > 0.05
Prepulse intensity × genotype	*F*[2,150] = 0.70	*p* > 0.05
Genotype × treatment	*F*[1,150] = 0.095	*p* > 0.05
Prepulse intensity × genotype × treatment	*F*[2,150] = 0.54	*p* > 0.05

### AAV-mediated augmentation of GR nuclear translocation affects PPI *via* 5-HT_2A_R

**Figure 10:**
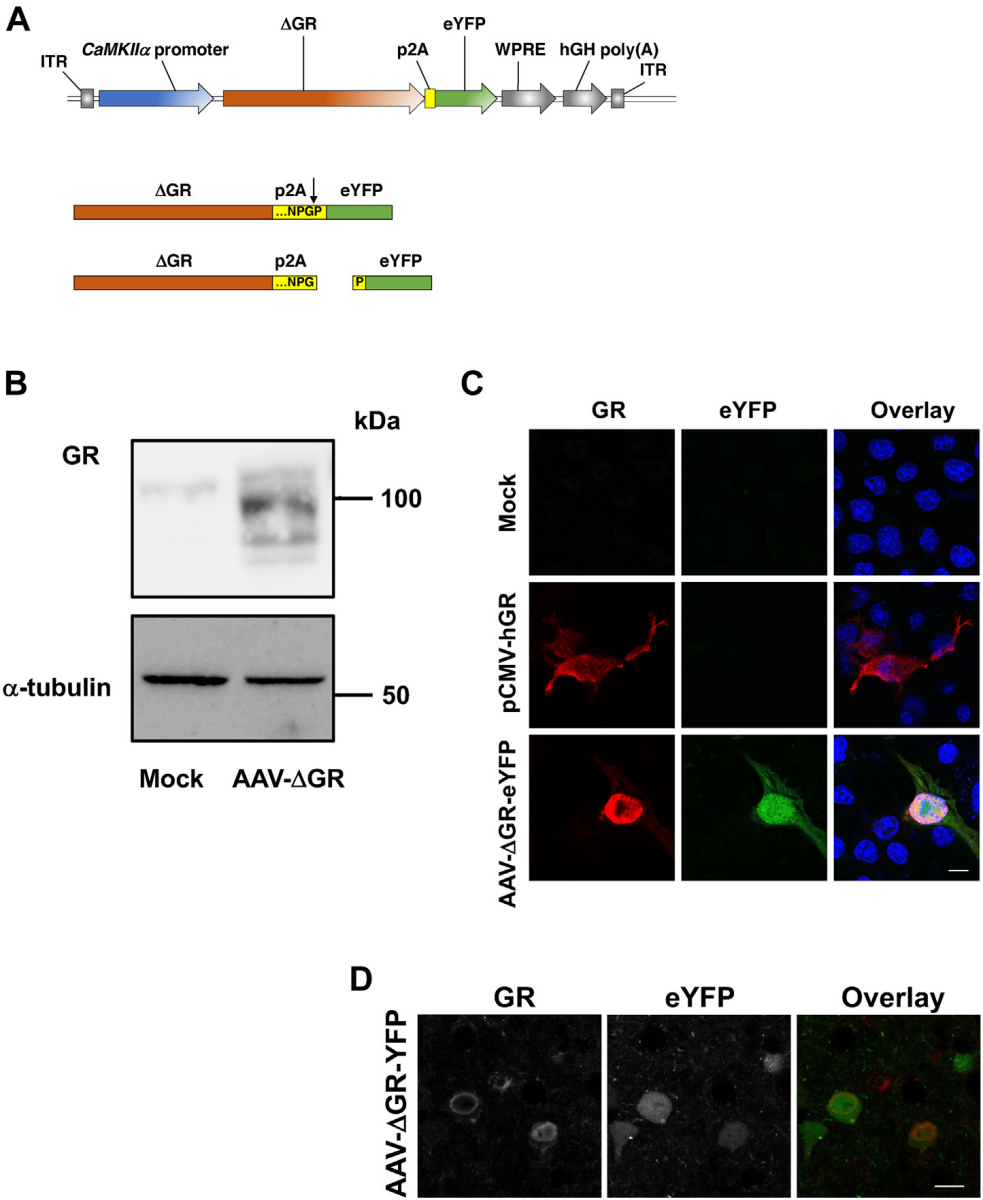
**Virally mediated augmentation of GR function.***A*, schematic representation of the recombinant AAV vector used to overexpress both ΔGR and eYFP under the *CaMKIIα* promoter in mouse frontal cortex pyramidal neurons. The a*rrowhead* indicates p2A cleave site. *B*, representative immunoblot of Neuro-2a cells transfected with mock or AAV-ΔGR-p2A-eYFP plasmids. *C*, representative immunocytochemical images of Neuro-2a cells transfected with mock, pCMV-hGR, or AAV-ΔGR-p2A-eYFP plasmids. *D*, representative immunohistochemical images of the mouse frontal cortex injected with the AAV-CaMKIIα::ΔGR-p2A-eYFP (AAV-ΔGR-eYFP) construct. Note that signals of ΔGR (*red*) and eYFP (*green*) do not overlap, indicating high cleavage efficacy of the p2A site (*C* and *D*). The scale bars represent 10 μm (*C* and *D*). AAV, adeno-associated virus; eYFP, enhanced YFP; GR, glucocorticoid receptor.

**Figure 11:**
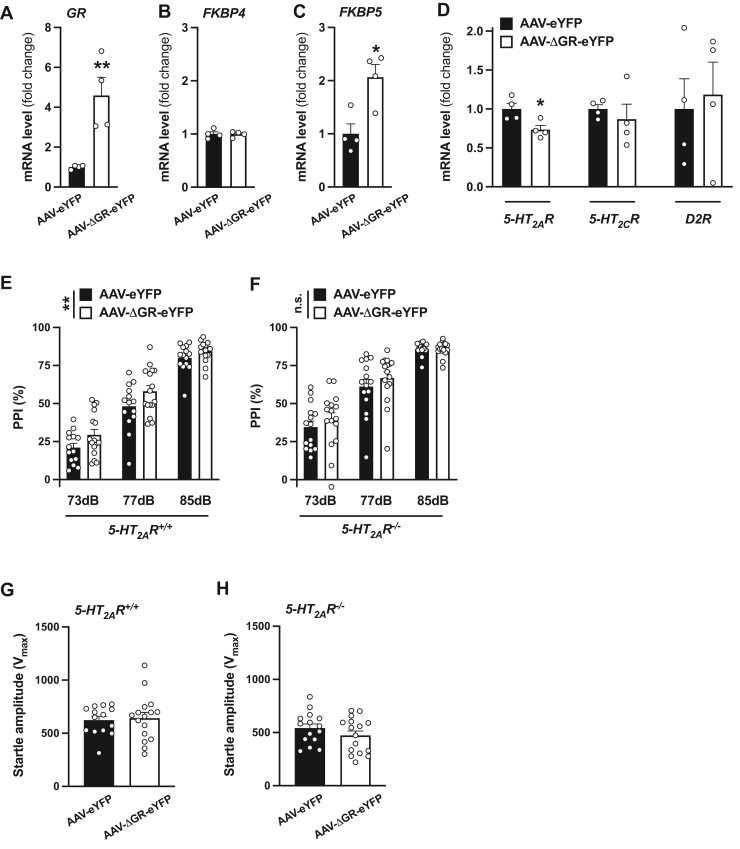
**Virally mediated augmentation of GR function downregulates frontal cortex *5-HT***_***2A***_***R* mRNA expression and disrupts sensorimotor gating in mice.***A*–*D*, AAV-ΔGR-eYFP or AAV-eYFP empty vector was injected into the frontal cortex. Mice were sacrificed for analysis 3 weeks after surgery, and expression of *GR* (*A*), *FKBP4* (*B*), *FKBP5* (*C*), and *5-HT*_*2A*_*R*, *5-HT*_*2C*_*R*, and *D*_*2*_*R* mRNAs (*D*) in frontal cortex was assessed by qRT-PCR (n = 4 male and female 129S6/SvEv mice/group). *E*–*H*, AAV-ΔGR-eYFP or AAV-eYFP empty vector was injected into the frontal cortex of *5-HT*_*2A*_*R*^*+/+*^ (n = 14–16 male 129S6/SvEv mice/group) and *5-HT*_*2A*_*R*^*−/−*^ (n = 15–16 male 129S6/SvEv mice/group) animals. %PPI (*E* and *F*) and startle amplitude (*G* and *H*) was tested 3 weeks after surgery. Data show mean ± SEM. ∗*p* < 0.05, ∗∗*p* < 0.01, ns, not significant. Unpaired two-tailed Student’s *t* test (*A: t*_6_ = 3.93, *p* < 0.01; *B*: *t*_6_ = 0.098, *p* > 0.05; *C*: *t*_6_ = 3.51, *p* < 0.05; *D*: *5-HT*_*2A*_*R t*_6_ = 2.93, *p* < 0.05; *5-HT*_*2C*_*R t*_6_ = 0.66, *p* > 0.05; *D*_*2*_*R t*_6_ = 0.32, *p* > 0.05; *G*: *t*_28_ = 0.27, *p* > 0.05; *H*: *t*_29_ = 1.22, *p* > 0.05). Two-way ANOVA followed by Bonferroni’s post hoc test (*E*: AAV-ΔGR-eYFP *versus* AAV-eYFP *F*[1,84] = 8.30, *p* < 0.01; prepulse intensity *F*[2,84] = 159.6, *p* < 0.001; interaction *F*[2,84] = 0.43, *p* > 0.05; *F*: AAV-ΔGR-eYFP *versus* AAV-eYFP *F*[1,87] = 1.32, *p* > 0.05; prepulse intensity *F*[2,87] = 92.77, *p* < 0.001; interaction *F*[2,87] = 0.36, *p* > 0.05; for three-way ANOVA, see Table 5). AAV, adeno-associated virus; *D*_*2*_*R*, dopamine D2 receptor; eYFP, enhanced YFP; GR, glucocorticoid receptor; *5-HT*_*2A*_*R*, serotonin 5-HT_2A_R receptor; PPI, prepulse inhibition; qRT, quantitative RT.

Systemically administered glucocorticoids exert a wide variety of physiological effects and pharmacological actions. To further test the functional significance of changes in frontal cortex GR-dependent transcriptional activity on 5-HT_2A_R expression, we next evaluated the effects of AAV-mediated gene transfer of a previously described GR construct (ΔGR) (57, 58), which lacks the hormone-binding domain and is therefore able to constitutively translocate to the nucleus where it has the potential to affect transcription. The ΔGR construct generated from mouse complementary DNA (cDNA) was subcloned into the AAV8 viral vector under the control of the *CaMKIIα* promoter, which, as mentioned previously, allows selective expression of the transgene in frontal cortex glutamatergic pyramidal, but not GABAergic inhibitory, neurons. In addition, the inclusion of the p2A peptide allows for expression of ΔGR and eYFP as independent proteins from the same transcript (59) (Fig. 10*A*). This was validated by immunoblot (Fig. 10*B*) and immunocytochemical (Fig. 10*C*) assays in Neuro-2a cells, which endogenously express the *CaMKIIα* gene, as well as immunohistochemical assays in mouse frontal cortex tissue sections (Fig. 10*D*). First, we confirmed that mice injected with the AAV-CaMKIIα:ΔGR-p2A-eYFP construct (AAV-ΔGR-eYFP) showed elevated expression of *GR* in the frontal cortex (Fig. 11*A*), as well as upregulation of *FKBP5* (Fig. 11*C*), but not *FKBP4* (Fig. 11*B*), mRNA expression. Notably, such overexpression of ΔGR decreased *5-HT*_*2A*_*R* mRNA but not *5-HT*_*2C*_*R* or *dopamine D_2_ receptor* mRNAs in this brain region (Fig. 11*D*).

To determine whether overexpression of ΔGR influences behavioral responses that involve 5-HT_2A_R-mediated signaling pathways, we tested the effects of AAV-ΔGR-eYFP compared with the empty AAV-eYFP vector on sensorimotor gating behavior in *5-HT*_*2A*_*R*^*+/+*^ and *5-HT*_*2A*_*R*^*−/−*^ mice. As before (Fig. 9, *D* and *E*, and Table 4), %PPI was increased in *5-HT*_*2A*_*R*^*−/−*^ animals as compared with *5-HT*_*2A*_*R*^*+/+*^ controls (Fig. 11, *E* and *F* and Table 5), whereas startle amplitude was similar in both groups of mice (Fig. 11, *G* and *H*). Notably, AAV-mediated overexpression of ΔGR in the frontal cortex increased %PPI behavior in *5-HT*_*2A*_*R*^*+/+*^ animals (Fig. 11*E*), whereas a similar manipulation of GR-dependent function failed to affect sensorimotor gating processes when this behavior model was tested in *5-HT*_*2A*_*R*^*−/−*^ mice (Fig. 11*F*).

**Table 5:** Three-way ANOVA of the effect of AAV-ΔGR-eYFP *versus* AAV-eYFP on %PPI in male 129S6/SvEv (in-house) mice

Source of variation	ANOVA	*p*
Prepulse intensity	*F*[2,171] = 241.5	*p* < 0.001
Viral vector	*F*[1,150] = 8.55	*p* < 0.01
Genotype	*F*[1,171] = 7.64	*p* < 0.001
Prepulse intensity × viral vector	*F*[2,171] = 0.78	*p* > 0.05
Prepulse intensity × genotype	*F*[2,171] = 1.71	*p* > 0.05
Genotype × viral vector	*F*[1,171] = 1.10	*p* > 0.05
Prepulse intensity × genotype × viral vector	*F*[2,171] = 0.0028	*p* > 0.05

**Figure 12:**
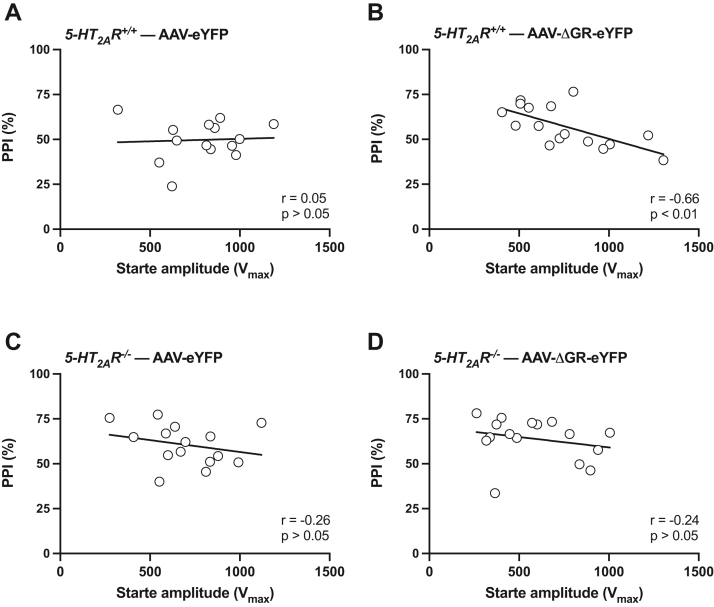
**AAV-dependent manipulation of GR function affects correlation between startle amplitude and sensorimotor gating *via* 5-HT**_**2A**_**R.***A*–*D*, effect of AAV-ΔGR-eYFP (*B* and *D*) *versus* AAV-eYFP (*A* and *C*) on Pearson’s correlation between startle magnitudes within the five stimulus-only trials at the beginning of the PPI session and %PPI in *5-HT*_*2A*_*R*^*+/+*^ (*A* and *B*) and *5-HT*_*2A*_*R*^*−/−*^ (*C* and *D*) mice (n = 14–16 male 129S6/SvEv mice/group) and *5-HT*_*2A*_*R*^*−/−*^ (n = 15–16 male 129S6/SvEv mice/group) animals. Correlation analysis was conducted using Pearson’s *r* (*A–D*). AAV, adeno-associated virus; eYFP, enhanced YFP; GR, glucocorticoid receptor; 5-HT_2A_R, serotonin 5-HT_2A_R receptor; PPI, prepulse inhibition.

We also tested whether AAV-dependent manipulation of GR transcriptional activity affected the correlation between the magnitude of the startle amplitudes during the five pulse-only trials at the beginning of the PPI session and the average %PPI in both genotypes. Importantly, frontal cortex injection of the AAV-ΔGR-eYFP construct led to a negative correlation between startle amplitudes prior to the PPI test and average %PPI in *5-HT*_*2A*_*R*^*+/+*^ mice (Fig. 12, *A* and *B*), an effect that was not observed in *5-HT*_*2A*_*R*^*−/−*^ littermates (Fig. 12, *C* and *D*). This suggests that virally mediated augmentation of frontal cortex GR function promotes behavioral sensorimotor gating processes *via* 5-HT_2A_R.

## Discussion

Prior to these studies, although 5-HT_2A_R dysregulation in frontal cortex of rodent MIA models had been demonstrated (28, 29, 30), the underlying cause of this alteration remained unsolved. Our data using three independent models suggest that alterations in GR signaling, including both decreased nuclear immunoreactivity and reduced GR binding to the *5-HT*_*2A*_*R* promoter, underlie increased *5-HT*_*2A*_*R* expression in MIA offspring mouse frontal cortex. Dysregulation of 5-HT_2A_R, in turn, was necessary for MIA-induced reduction in mature mushroom frontal cortex dendritic spine density—implicating this process of synaptic structure alterations in the MIA model. Together, these data provide key insights into mechanisms leading to 5-HT_2A_R dysregulation in the context of prenatal insults, clarifying the GR-dependent control of *5-HT*_*2A*_*R* abnormal expression and elucidating potential mechanisms involved in synaptic and sensorimotor gating deficits within the preclinical model.

One of the most intriguing findings of our study was the potential repressive role of the transcription factor GR in the promoter activity of the *5-HT*_*2A*_*R* gene. Using ChIP assays in frontal cortex samples from naïve mice, we showed enrichment of GR binding at a region of the *5-HT*_*2A*_*R* gene containing a putative GR-binding site. However, in frontal cortex samples of adult mice born to mothers injected with poly-(I:C) during pregnancy, we found a significantly decreased enrichment of GR at the *5-HT*_*2A*_*R* gene promoter. This MIA-dependent epigenetic modification was associated with augmentation of *5-HT*_*2A*_*R* mRNA transcription in the mouse frontal cortex, which suggests that GR plays a repressive role in the transcriptional activity of the *5-HT*_*2A*_*R* gene. This concept is further supported by our findings suggesting downregulation of *5-HT*_*2A*_*R* expression upon continued activation of the GR pathway *via* AAV-ΔGR, which provides a crucial advancement to work on the relationship between the two receptors, and yields information about a potential mechanism for the exquisite sensitivity of 5-HT_2A_Rs to stress.

The relationship between stress and frontal cortex *5-HT*_*2A*_*R* expression and related phenotypes is a robustly demonstrated phenomenon, observed following a variety of stressors, such as methamphetamine binge (60), maternal separation (61), and sleep deprivation (62). It has been speculated that this relationship between stress and the *5-HT*_*2A*_*R* serves as a mechanism by which organisms under stress can upregulate a receptor associated with synaptic plasticity, potentially altering behavior in an advantageous manner (63, 64). In the context of gestational insults in mouse (28, 29, 30, 31, 34, 35) and rat (32, 33) MIA models, upregulation of frontal cortex *5-HT*_*2A*_*R* expression may be maladaptive and instead promote phenotypes related to perception and sensory processing. Interestingly, increased *FKBP5* mRNA has been reported in postmortem prefrontal cortex samples from an Australian cohort of schizophrenia subjects relative to controls (65), whereas 5-HT_2A_R density is upregulated in frontal cortex of samples from subjects with schizophrenia (24, 25, 26, 27)—suggesting that certain components of the GR-5-HT_2A_R pathway are dysregulated within the human disorder. We previously reported upregulation of frontal cortex 5-HT_2A_R and augmentation of psychedelic-induced head-twitch behavior in adult, but not prepubertal, MIA offspring as compared with controls (29, 30), which supports construct validity of our model of neurodevelopmental psychiatric conditions since, as an example, the onset of schizophrenia usually occurs during adolescence or early adulthood (1). While we have demonstrated an association between decreased GR enrichment at the *5-HT*_*2A*_*R* promoter (a more proximal evaluation of GR interaction with the *5-HT*_*2A*_*R* promoter than GR protein in the nuclear compartment) with increased *5-HT*_*2A*_*R* expression in two completely independent experimental systems, as well as decreased *5-HT*_*2A*_*R* expression with direct brain region–specific GR overexpression, further work needs to be completed to clarify the mechanisms by which GR dysregulation itself occurs within MIA models. Similarly, follow-up work could explore both the timing of these dysregulations throughout prenatal and postnatal development and adulthood as well as the level at which GR dysregulation occurs. As mentioned previously, there is a well-established relationship between GR signaling and *5-HT*_*2A*_*R* expression and related phenotypes. To our knowledge, our work is the first to explore this associated relationship within the context of an MIA model.

Although sex-related differences are not the main objective of this study and consequently not all the experimental techniques were confirmed in both sexes, our data suggest that MIA has a primarily sex-independent effect on two key alterations in the adult offspring (PPI deficits and density of mature mushroom spines). However, our data also suggest that dendritic structural elements such as density of immature thin spines and total spine density may vary among sexes. Further investigation will be focused on a full exploration of sex-specific differences related to MIA models.

Regarding validation of our MIA model, our data suggested induction of IL-6 and weight loss in nonpregnant female mice following poly-(I:C) administration. It has also been proposed that maternal SFB are necessary for induction of maternal IL-17A (43), which is necessary for at least part of the MIA-induced phenotypes (66). Using qPCR on *16S ribosomal RNA* from murine cecal contents, we demonstrate the presence of SFB in the gut of mice from sources used for our studies, but not in mice from JAX, which have previously been shown to lack gut SFB and whose offspring did not exhibit MIA-induced phenotypes following maternal poly-(I:C) administration (43). While this supports the validity of our experimental design, we also recognize that the stress of shipping could contribute to maternal stress and potentially impact offspring phenotypes.

Our current data raise the question of why repeated administration of systemic corticosterone and local frontal cortex AAV-ΔGR injection exert opposite effects on both *5-HT*_*2A*_*R* mRNA expression and %PPI. While these studies were all conducted in male 129S6/SvEv mice (Table 1), one possible explanation is the ability of negative feedback to constrain GR-dependent signaling. Although both repeated corticosterone and ΔGR result in increased *FKBP5* expression, suggesting the existence of negative feedback within both experimental systems, AAV-ΔGR injection results in a roughly fourfold, at least at the mRNA level, induction of *GR* expression. In addition, the ΔGR truncation is capable of constitutive nuclear translocation and is not expressed under the control of the endogenous *GR* locus, both of which could render its resistance to endogenous negative feedback mechanisms. Our observation that repeated corticosterone administration results in decreased enrichment of the GR at the *5-HT*_*2A*_*R* promoter further supports the hypothesis that negative feedback may explain this discrepancy. Thus, sustained AAV-mediated ΔGR occupancy at the *5-HT*_*2A*_*R* promoter would suppress *5-HT*_*2A*_*R* transcription, whereas negative feedback upon repeated corticosterone administration would reduce endogenous GR occupancy of the *5-HT*_*2A*_*R* promoter and allow stimulation of *5-HT*_*2A*_*R* expression. In addition, while ΔGR-induced PPI improvements were absent in *5-HT*_*2A*_*R*^*−/−*^ mice, corticosterone-induced PPI deficits were observable in both genotypes. While this might be explained by the systemic nature of corticosterone administration, in contrast to the frontal cortex–specific AAV-mediated ΔGR expression, further studies are needed to clarify what cellular target(s) and molecular changes underlie sensorimotor gating alterations induced by this model of repeated pharmacological activation of the GR.

We report that MIA-induced alterations in mushroom dendritic spine density are 5-HT_2A_R dependent. Of note, *5-HT*_*2A*_*R*^*−/−*^ mice exhibited lower mature mushroom spine density compared with *5-HT*_*2A*_*R*^*+/+*^ controls regardless of the maternal treatment. Within a different experimental paradigm, we have recently observed similar decreases in frontal cortex spine density in *5-HT*_*2A*_*R*^*−/−*^ mice as compared with *5-HT*_*2A*_*R*^*+/+*^ controls (50). Given the implication of ligands that activate (50, 67) or block (49) 5-HT_2A_R-dependent signaling in processes related to synaptic structural and functional plasticity, our findings corroborate the fundamental role of this serotonin receptor in these processes. Follow-up studies building on this work, such as overexpressing ΔGR and/or 5-HT_2A_R in the frontal cortex of MIA and control offspring to determine if these manipulations are sufficient to affect dendritic spine density, could further explore this relationship and would be useful for confirming the mechanisms suggested by the data presented here.

Our data support a negative regulatory relationship between GR-dependent signaling and 5-HT_2A_R expression with implications for dendritic structural plasticity, functional GR nuclear translocation, and behavior phenotypes observed in mouse MIA models. These findings advance our understanding of the molecular mechanisms by which prenatal insults upregulate 5-HT_2A_R expression in the frontal cortex of the adult offspring.

## Experimental procedures

### Materials, drug administration, and mouse brain and blood samples

Poly-(I:C) (MilliporeSigma; catalog no.: P9582) was dissolved in normal saline (0.9% NaCl) and, after a brief centrifugation (800*g*), the resulting solution (4 mg/ml) was aliquoted and stored at −20 °C until the day of the experiment.

Corticosterone (MilliporeSigma; catalog no.: 46148) was diluted in dimethyl sulfoxide (DMSO) (100 mg/ml), and aliquots were stored at −20 °C until the day of the experiment. Adult male mice were injected (s.c.) with corticosterone (50 mg/kg) dissolved in a final solution of DMSO:ethanol (1:3). For time-course assays, all mice (excluding the no-injection group) were injected (s.c.) twice a day over 8 days with corticosterone (50 mg/kg), or vehicle, and sacrificed 8.5 to 13 h after the last injection. For the short-term experiments, adult male mice received (s.c.) corticosterone (50 mg/kg), or vehicle, twice a day for 4 days. Mice were either sacrificed or tested for behavior 8.5 to 13 h after the last injection.

For brain samples, mice were sacrificed for analysis by cervical dislocation, and bilateral frontal cortex (bregma 1.40–1.90 mm) was dissected and either frozen at −80 °C or immediately processed for RNA extraction, ChIP, and/or biochemical assays. The coordinates were taken according to a published atlas of the C57BL/6 and 129Sv mouse brains (68).

For serum samples, following decapitation, trunk blood was collected and allowed to clot at room temperature for at least 30 min. The blood was then centrifuged (1000*g*) at 4 °C for 10 min after which the serum supernatant was collected and stored at −80 °C until the day of the assays.

All other chemicals were obtained from standard sources.

### Animals

Experiments were performed on adult (10–22 week old) male C57BL6/N, C57BL6/J, and/or 129S6/SvEv mice (Table 1). Animals were housed on a 12 h light/dark cycle (lights on 6 AM to 6 PM) at 23 °C with food and water ad libitum, except during behavioral testing, which took place during the light cycle. All procedures were conducted in accordance with the National Institutes of Health (NIH) guidelines and were approved by the Virginia Commonwealth University Animal Care and Use Committee. Behavioral testing took place between 9:00 AM and 6:00 P.M.

5-HT_2A_R (*Htr2a*) knockout (*5-HT*_*2A*_*R*^*−/−*^) mice on a 129S6/SvEv background have been previously described (69). For experiments involving *5-HT*_*2A*_*R*^*−/−*^ mice, wildtype (*5-HT*_*2A*_*R*^*+/+*^) littermates on a 129S6/SvEv background were used as controls. All subjects were offspring of heterozygote breeding.

### Immune activation with poly-(I:C)

To test the effect of poly-(I:C) administration on female immune response (data presented in Fig. 1), naïve adult animals (C57BL6/N from CRL and 129S6/SvEv generated *via* in-house breeding) were injected (i.p.) with poly-(I:C) (20 mg/kg), or vehicle, and observed for sickness behavior, such as lethargy, ptosis, and hunched posture. After 2.5 h, mice were sacrificed by either decapitation and exsanguination (for experiments involving serum samples) or by cervical dislocation for collection of frontal cortex samples.

To test the effect of MIA during pregnancy (data presented in Figs. 3, 5, 6, *D*, *E*, and 7, *A–D*), pregnant (E12.5) female mice were injected (i.p.) with poly-(I:C) (20 mg/kg), or vehicle, and then monitored for sickness behavior. This timing was chosen because the first and second trimesters have been shown to be a critical period for increased risk of neurodevelopmental conditions including schizophrenia and autism following influenza virus infection in humans (8), and MIA at E12.5 has been demonstrated to produce MIA-induced phenotypes in offspring (17). To reduce litter loss, MIA or mock mice were pair-housed with another nonpregnant female of the same strain and provided with enrichment Bio-Huts (Bio-Serv) as shelter in the cage. It has been shown that litter effects are substantial (70). To avoid litter effects, animals from at least two separate litters were subjected to the different protocols (Table 1). For MIA experiments in “wild-type” animals, assays were carried out with either timed pregnant C57BL6/N mice ordered from CRL (Figs. 3, *A*, *B*, 6, *D*, *E*, and 7, *A–D*) or C57BL6/N females in-house bred with C57BL6/N males from CRL (Fig. 3, *C* and *D*). For MIA experiments in *5-HT*_*2A*_*R*^*−/−*^ mice and *5-HT*_*2A*_*R*^*+/+*^ controls (Fig. 5), 129S6/SvEv female *5-HT*_*2A*_*R*^*+/−*^ (heterozygote) mice (Taconic background) were bred in-house; females were monitored for mating plug, and pregnancy was evaluated using both weight gain and visual appearance of the flanks. All offspring (C57BL6/N and 129S6/SvEv) were weaned between 3 and 4 weeks of age, and littermates were evaluated in biochemical, dendritic spine structure, and/or behavioral assays as adult animals (10–22 weeks old).

### PPI of startle

PPI of startle experiments were conducted following a previously described paradigm (71). Briefly, mice were placed in SR-Lab Startle Response System (San Diego Instruments) chambers and allowed to habituate to background noise for 5 min. Following habituation, mice were exposed to five startle-only trials. They were then subjected to 65 pseudorandomized trials consisting of interspersed no-stimulus trials, startle-only trials, or 73 dB, 77 dB, or 85 dB prepulse trials. The session concluded with five additional startle-only trials. %PPI was calculated based on averages for each trial type during those 65 trials by the equation %PPI = (1 – [*V*_max_ of prepulse trials/*V*_max_ of stimulus-only trials]) × 100. Startle magnitude was calculated as the average of the *V*_max_ value for the five initial startle-only trials. Background noise was maintained at 69 dB for the duration of the experiment. The startle-inducing stimulus was a 120 dB pulse lasting for 20 ms. For prepulse trials, a 20 ms prepulse of 73 dB, 77 dB, or 85 dB was administered prior to the startle stimulus. The interstimulus interval was 100 ms from the start of the prepulse to the start of the startle stimulus. The intertrial interval was an average of 15 s, with a range of 12 to 18 s. Each PPI session lasted a total of about 30 min.

### Plasmid construction

The AAV-CaMKIIα::eYFP construct has been described previously (49). The pCMV-GR11 construct was obtained from Addgene (catalog no.: 89105). To generate the AAV-CaMKIIα::ΔGR-p2A-eYFP construct, a PCR fragment was obtained from mouse frontal cortex cDNA as a template using the primers mΔGR-BamHI/S and mΔGR-Ascl/A (mΔGR cloning forward and reverse, respectively; Table S6). The resulting PCR product was digested with BamHI and AscI and subcloned into the same restriction sites of AAV-CaMKIIα-c-Myc-5-HT_2A_R-p2A-eYFP. All PCR construct amplifications were performed with *PfuUltra* high-fidelity DNA polymerase (Agilent) in a Mastercycler EP Gradient Auto thermal cycler (Eppendorf). All the constructs were confirmed by DNA sequencing.

### Virally mediated gene transfer

The construct vectors CaMKIIα::eYFP and CaMKIIα::ΔGR-p2A-eYFP were packaged into AAV serotype 8 vector particles, all produced by the University of North Carolina at Chapel Hill Vector Core. The AAV-eYFP and AAV-ΔGR-eYFP construct vectors were injected into the frontal cortex (+1.6 mm rostrocaudal, −2.4 mm dorsoventral, and +2.6 mm mediolateral relative to bregma) of adult mice by stereotaxic surgery according to standard methods (49, 50). Mice were anesthetized with isoflurane (2% initial dose, 1–2% maintenance) during the surgery.

### Dendritic spine analysis

Frontal cortex dendritic spine structural assays were performed as previously reported (49, 50). Briefly, male and female mice were deeply anesthetized (ketamine/xylazine) after which transcardial perfusion was performed with PBS (about 10 ml) followed by 30 ml of 4% paraformaldehyde (PFA) in PBS. Following perfusion, the brain was dissected and stored in 30% sucrose in PBS at 4 °C until sectioning. The frontal cortex samples were cut into 50 μm sections (VT1000S vibratome; Leica) after which sections were stored at 4 °C until immunofluorescence. For immunofluorescence assays, frontal cortex tissue sections were incubated in 4% PFA in PBS for 20 min. Sections were then washed with PBS for 10 min and permeabilized in 0.1% Triton X-100 in PBS for 15 min. After an additional two washes in PBS, sections were blocked in blocking buffer (5% bovine serum albumin [BSA] in 0.1% Triton X-100 in PBS). Sections were then incubated with primary antibody (rabbit anti-GFP; Invitrogen; catalog no.: A-11122, 1:1000 dilution) in blocking buffer overnight at 4 °C. The sections were then washed in PBS and incubated with secondary antibody (anti-rabbit; Alexa-488, Invitrogen A-11008; 1:2000 dilution) in blocking buffer at room temperature for 1 h. After this, the sections were washed three times with PBS and mounted on #1.5 coverslips (Fisher) in ProLong Diamond antifade mountant (Invitrogen). After placement of slides, the sections were allowed to cure overnight before sealing with nail polish top coat (Seche Vite). Slides were stored at 4 °C in the dark until imaging.

Slides were imaged by confocal laser scanning microscopy using the 488 nm laser line of an LSM 710 microscope (Zeiss). Z-stacks were acquired for layer 5 dendrites in mouse frontal cortex using a 63× objective with 2.5× zoom, yielding stacks of 632 × 632 pixel images with a voxel size of 0.09 μm × 0.09 μm × 0.2 μm. Files were converted to TIFF format for analysis. Dendrite image stacks were then analyzed using NeuronStudio (a non-commercial software created at Mount Sinai School of Medicine [49, 50]) by a blinded experimenter. Dendritic lengths were determined by tracing the dendrite images within the software. With its Rayburst sampling algorithm, NeuronStudio was used to classify dendritic spines as stubby, thin, or mushroom in morphology.

### Transient transfection of Neuro-2a cells

Neuro-2a cells (American Type Culture Collection; CCL-131) were maintained in a growth medium of Dulbecco's modified Eagle's medium with glucose, l-glutamine, and sodium pyruvate (Corning) supplemented with 5% qualified fetal bovine serum (Gibco) and 1% 10,000 U/ml penicillin/10,000 μg/ml streptomycin solution (Gibco) at 37 °C in a 5% CO_2_ humidified atmosphere. To prepare a surface for adherence of Neuro-2a cells, coverslips (#1) (Fisherbrand) were incubated in 10 M NaOH at room temperature overnight with gentle shaking, washed, and autoclaved. The coverslips were coated in poly-d-lysine (Sigma–Aldrich) in borate buffer (50.1 mM BH_3_O_3_ and 12.5 mM Na_2_B_4_O_7_·10H_2_O) overnight. The next day, coverslips were washed three times in sterile water and then allowed to dry. For transfection, Neuro-2a cells were plated on poly-d-lysine-coated coverslips and allowed to grow. Neuro-2a cells on coverslips in 6-well plates were transfected with 4 μg of plasmid DNA with 5 μl of polyethyleneimine made up to a final volume of 300 μl with Opti-MEM reduced serum medium (Gibco) that were added to growth medium. Prior to transfection, DNA, polyethyleneimine, and Opti-MEM were combined, shaken, and incubated for 10 min at room temperature. The medium was replaced with regular growth medium the day after transfection. Cells were processed for immunofluorescence 48 h after transfection as described later. For immunoblot assays, Neuro-2a cells in 10 cm plates were transfected with 6 μg of plasmid DNA (AAV-ΔGR-eYFP plasmid construct, see above, or a construct expressing the full-length human GR under control of the cytomegalovirus promoter, pCMV-GR11; Addgene; catalog no.: 89105) with 10 μl of polyethyleneimine made up to a final volume of 300 μl with Opti-MEM as described above. To harvest, transfected Neuro-2a cells were washed with Dulbecco’s PBS three times before being dislodged with a cell scraper in cold Dulbecco’s PBS. Cells were then pooled and spun down for 5 min at 188*g* at room temperature. The supernatant was removed, and pelleted cells were frozen at −80 °C until they were subjected to subcellular fractionation followed by immunoblot assays as described later.

### Immunohistochemistry

In Neuro-2a cells, experiments were performed as previously reported with minor modifications (49). Briefly, GR immunoreactivity was assayed by using an anti-GR antibody (Santa Cruz; catalog no.: sc-393232; 1:50 dilution) and Alexa 568 dye-conjugated anti-mouse secondary antibody (Invitrogen; catalog no.: A-11004; 1:1000 dilution).

In mouse frontal cortex, brains of 39-week-old female mice that had been stereotaxically injected with AAV-eYFP *versus* AAV-ΔGR-eYFP were cut into 50 μm sections, and the resulting slices were then subjected to the immunofluorescence staining protocol (see above): primary antibody (mouse anti-GR; Santa Cruz; catalog no.: sc-393232; 1:50 dilution), secondary antibody (anti-mouse Alexa-488; Invitrogen; catalog no.: A-11004; 1:2000 dilution). Images were acquired using the 405, 488, and 561 nm laser lines of an LSM 710 confocal laser scanning microscope. A 63× objective was used, resulting in 1912 × 1912 pixel images with a 0.07 μm × 0.07 μm pixel size.

### FISH

Using a subcloned and sequenced PCR fragment (162 bp; Table S6) of the mouse *5-HT*_*2A*_*R* cDNA (Zero Blunt TOPO PCR Cloning kit; Invitrogen), high-specific activity RNA probes were produced from 3 μg of linearized plasmid (HindIII) and labeled using the Fluorescein RNA Labeling Mix (Roche), following the manufacturer's instructions. All work surfaces and dissection tools were thoroughly cleaned with RNAse Zap (Life Technologies), and great care was taken to maintain an RNase-free environment during tissue sample collection. Adult mice were perfused, and brain samples were sectioned as described previously with minor modifications that included transcardial perfusion with diethyl pyrocarbonate (DEPC)-PBS (about 10 ml) followed by 30 ml of 4% microscopy grade PFA (Electron Microscopy Sciences) in DEPC-PBS at 4 °C. Following perfusions, the brain was dissected and stored in cryoprotectant (2% DMSO and 20% glycerol in DEPC-PBS) until sectioning. The frontal cortex samples were cut into 20 μm sections (VT1000S vibratome) after which sections were stored at 4 °C until hybridization. Coronal brain sections were washed with DEPC-PBS and incubated with saline sodium citrate (SSC) 5× buffer (748 mM NaCl, 74.8 mM Na_3_C_6_H_5_O_7_·H_2_O in DEPC-H_2_O, pH 7) with shaking. A border was drawn around the sections with a Liquid Blocker hydrophobic pen (Daido Sangyo Co), and slides were placed in a preheated humidified StainTray Slide Staining System (Simport). The slides were covered with blocking solution (50% formamide, 25% SSC 20× buffer [2.99 M NaCl, 299 mM Na_3_C_6_H_5_O_7_·H_2_O in DEPC-H_2_O, pH 7.0], 25% DEPC-H_2_O, 0.8% salmon sperm DNA; Invitrogen) and incubated at 37 °C for 2 h. The fluorescein-labeled RNA probe was diluted at the appropriate concentration (1:20–1:100) in hybridization buffer (50% formamide, 40% dextran sulfate solution; Millipore, 10% SSC 20×), and the resulting solution was heated at 77 °C for 5 min. The blocking solution was removed from the sections, and the probe in hybridization buffer was added; the slides were then covered with HybriSlip hybridization covers (Grace Bio-Labs), sealed with Fixogum rubber cement (Marabu), and incubated overnight at 37 °C. The next day, the slide covers were removed and slides were washed at room temperature in SSC 2× buffer (299 mM NaCl, 29.9 mM Na_3_C_6_H_5_O_7_·H_2_O in ddH_2_O, pH 7.0) for 5 min three times in Coplin jars with shaking. The slides were then washed for 30 min with shaking in SSC 2× buffer that had been heated to 43 °C, after which the slides were washed again for 30 min with shaking in SSC 0.1× buffer (15 mM NaCl, 1.5 mM Na_3_C_6_H_5_O_7_·H_2_O in ddH_2_O, pH 7.0) that had been heated to 43 °C. Autofluorescence was then reduced by incubating slides with shaking in Coplin jars containing 0.1% Sudan Black B that had been dissolved in 70% ethanol in the dark overnight and syringe filtered using a 0.45 μm filter (Corning). Slides were then washed three times in PBS with shaking. Sections were dried, mounted in Duolink *In Situ* Mounting Medium with 4′,6-diamidino-2-phenylindole (Sigma–Aldrich), covered with #1.5 coverslips, and sealed. The 488 nm laser line of an LSM 710 confocal laser scanning microscope was used to acquire images from mouse frontal cortex (delineated by +1.4 to +1.9 mm relative to bregma). Images were acquired with a 63× objective, yielding 1912 × 1912 pixel images with a pixel size of 0.07 μm × 0.07 μm. Files were converted to TIFF format for analysis. Fluorescence intensity was evaluated by a blinded experimenter using ImageJ (NIH): a region of interest was drawn around the border for each cell, and mean fluorescence intensity in the region of interest was used for data analysis—cells were selected from among two fields from each animal.

### ELISAs

ELISAs were performed using the mouse IL-6 ELISA-MAX kit (BioLegend) using the manufacturer’s instructions. Briefly, 100 μl of a 1:200 dilution of capture antibody in coating buffer (100 mM NaHCO_3_, 33.6 mM Na_2_CO_3_ in ddH_2_O, pH 9.5) was added to wells of a 96-well Nunc MaxiSorp plate (Invitrogen) and incubated at 4 °C overnight. The next day, the wells were washed in 300 μl wash buffer (137 mM NaCl, 2.68 mM KCl, 10.1 mM Na_2_HPO_4_, 1.76 mM KH_2_PO_4_, 0.05% Tween-20, pH 7.4) three times. The wells were then blocked in 200 μl assay diluent (137 mM NaCl, 2.68 mM KCl, 10.1 mM Na_2_HPO_4_, 1.76 mM KH_2_PO_4_, 1% BSA in ddH_2_O, pH 7.4) at room temperature for 1 h with shaking. The wells were then washed four times in wash buffer. After this, 100 μl of 1:100 dilutions of serum samples in assay diluent were added to wells in triplicate, as were mouse IL-6 standards (7.8–500 pg/ml) diluted in assay diluent, before sealing and incubation with shaking at room temperature for 2 h. The wells were then washed four times in wash buffer after which 100 μl of a 1:200 dilution of detection antibody in assay diluent was added to each well and the plate was sealed and incubated at room temperature for 1 h with shaking. The wells were then washed four times in wash buffer before addition of 100 μl of a 1:1000 dilution of Avidin-horseradish peroxidase (HRP) in assay diluent, sealing, and incubation at room temperature for 30 min with shaking. The plate was then washed five times in wash buffer, with soaking of wells for 30 s to 1 min during each wash. After this, 100 μl of TMB substrate solution (BioLegend, a 1:1 mixture of TMB substrate A and TMB substrate B) was added to each well and incubated in the dark for 5 to 10 min. The reaction was stopped by addition of 100 μl stop solution (BioLegend), and absorbance was read at 450 nm with subtraction of the absorbance at 570 nm using a VICTOR Nivo multimode plate reader (PerkinElmer).

### Quantitative real-time PCR

Real-time qPCR (qRT-PCR) assays were carried out as previously described. Analysis was done using the ΔΔC_T_ method (72). Primer pair sequences are listed in Table S6—see also (43, 49, 73, 74, 75, 76, 77).

### Microbiome evaluation

Adult female mice (C57BL6/N from CRL, C57BL6/N from Taconic, C57BL6/J from JAX, and 129S6/SvEv generated *via* in-house breeding) were sacrificed, and cecum samples were collected based on previously described protocols (28). Briefly, Eppendorf tubes filled with cecum contents were then weighed to obtain wet weights, and samples were frozen at –80 °C until use. DNA was isolated using a PowerFecal DNA Isolation kit (MO BIO Laboratories), and DNA samples were then stored at −80 °C until use. After this, DNA samples (20 ng) were loaded into each reaction for qPCR (for primer pair sequences, see Table S6). Values are reported based on amplification of the 16S rRNA gene normalized to wet weight of cecum contents.

### Cellular fractionation

Neuro-2a cells, mouse frontal cortex, or postmortem human frontal cortex samples were homogenized in 1 ml of Tris–HCl (50 mM, pH 7.4) supplemented with 0.25 M sucrose (Neuro-2a and mouse) or 6 ml of Tris–HCl (50 mM, pH 7.4) supplemented with 0.32 M sucrose (postmortem human frontal cortex) using a Teflon-glass homogenizer. The samples were then centrifuged at 1000*g* for 5 min (mouse frontal cortex) or 15 min (postmortem human frontal cortex) at 4 °C. The supernatant (S1 fraction) was transferred to a new tube. The pellet (P1 fraction) was resuspended in Tris–HCl and centrifuged under the same conditions. The final pellet was resuspended in Tris–HCl and transferred to a new tube for use as the nuclear fraction. The S1 fraction was centrifuged at 40,000*g* for 20 min at 4 °C. The resulting supernatant (S2 fraction) was retained as the cytoplasmic fraction. The cytoplasmic and nuclear fractions were stored at −80 °C.

### Immunoblot assays

Western blot experiments were performed as previously reported, with minor modifications (49). Briefly, samples were loaded onto polyacrylamide gel (12%) and submitted to sodium dodecyl sulfate-PAGE. After transfer to nitrocellulose membranes, samples were blocked with 2.5% nonfat dry milk and 0.5% BSA in TBST buffer (65 mM Tris–HCl, 150 mM NaCl, 0.05 Tween-20, pH 7.6), followed by overnight incubation with primary antibody at 4 °C or 1 h at room temperature. In Neuro-2a cells and mouse frontal cortex samples, the following primary antibodies were used: mouse anti-GR (Santa Cruz; catalog no.: sc-393232, 1:100 dilution), mouse anti-α-tubulin (Abcam; catalog no.: ab7291, 1:3000 dilution), rabbit anti-β-actin (Abcam; catalog no.: ab8227, 1:3000–1:20,000 dilution). In Neuro-2a cells and mouse frontal cortex samples, incubation with secondary antibodies (HRP-linked mouse IgG; Amersham, catalog no.: NA931, or HRP-linked rabbit IgG; Amersham, catalog no.: NA934, 1:5000 dilution) was performed at room temperature for 60 to 90 min, followed by repeated washing with TBST. Immunoreactive proteins were visualized using a ChemiDoc MP Imaging System (Bio-Rad). In each case, the blots were stripped and reprobed for a control protein to control loading amounts. All immunoblots were quantified by densitometry using ImageJ (NIH). In postmortem frontal cortex samples, incubation with secondary antibodies (Alexa 680-conjugated anti-mouse IgG, Invitrogen; catalog no.: A21057, 1:6000 dilution, or DyLight 800-conjugated anti-rabbit IgG, Rockland, catalog no.: 611-745-127; 1:10,000 dilution) was performed at room temperature for 60 to 90 min, followed by repeated washing with TBST. Immunoreactive proteins were visualized using an Odyssey infrared imaging system (LI-COR Biosciences). Signal was quantified as integrated intensity using Image Studio Lite version 5.2 (LI-COR Biosciences).

### ChIP assay

ChIP experiments were performed as previously reported with minor modifications (49, 72), using a MAGnify Chromatin Immunoprecipitation System (Invitrogen). Primer pair sequences are listed in Table S6.

### Postmortem human brain samples

Human brains were obtained at autopsies performed in the Basque Institute of Legal Medicine, Bilbao, Spain. The study was developed in compliance with policies of research and ethical review boards for postmortem brain studies (Basque Institute of Legal Medicine, Spain). Retrospective searches were conducted for previous medical diagnosis and treatment using examiners’ information and records of hospitals and mental health centers. After searching antemortem information, 32 subjects who had met criteria of schizophrenia according to the Diagnostic and Statistical Manual of Mental Disorders (DSM-IV and/or DSM-IV TR) were selected (78). Toxicological screening for antipsychotics, other drugs, and ethanol was performed in blood, urine, liver, and gastric content samples. The toxicological assays were performed at the National Institute of Toxicology, Madrid, Spain, using a variety of standard procedures including radioimmunoassay, enzymatic immunoassay, high-performance liquid chromatography, and gas chromatograph mass spectrometry. The schizophrenia subjects were divided into 16 antipsychotic-free (AP-free) and 16 antipsychotic-treated (AP-treated) subjects according to the presence or the absence of antipsychotics in blood at the time of death. Controls for the present study were chosen among the collected brains on the basis, whenever possible, of the following cumulative criteria: (i) negative medical information on the presence of neuropsychiatric disorders or drug abuse and (ii) appropriate sex, age, postmortem delay (time between death and autopsy), and freezing storage time to match each subject in the schizophrenia group. Causes of death among the schizophrenia patients included suicide (n = 19), accidents (n = 2), and natural deaths (n = 11). Controls’ causes of death included accidents (n = 20) and natural deaths (n = 12). Specimens of prefrontal cortex (Brodmann’s area 9) were dissected at autopsy (0.5–1.0 g tissue) on an ice-cooled surface and immediately stored at −80 °C until use. Tissue pH values were within a relatively narrow range (control subjects: 6.48 ± 0.08; schizophrenia subjects: 6.28 ± 0.05). The definitive pairs of AP-free schizophrenia subjects and respective matched controls are shown in Table 2, and the definitive pairs of AP-treated schizophrenia subjects and respective matched controls are shown in Table 3. Pairs of schizophrenia patients and matched controls were processed simultaneously and under the same experimental conditions.

### Statistical analysis

Animals were randomly allocated into the different experimental groups. Statistical significance was assessed by Student’s *t* test, and one-way or two-way (or multiway) ANOVA, depending upon the number of experimental conditions and independent variables. Following a significant ANOVA, specific comparisons were made using the Bonferroni’s post hoc test. For the corticosterone time course, Dunnett’s post hoc test was used because the goal was to determine which groups were different from vehicle. Male and female mice were tested in Figures 3, *C*, *D*, 5, *B–F*, and 11, *A–D* (Table 1). For sex-related effects in Figures 3, *C*, *D* and 5, see Tables S1–S5. Sex as an independent variable was not tested in Figure 11, *A–D* because two mice per sex per group were utilized. Datapoints were excluded based on previously established criterion and were set to ±2 SD from the group mean. Correlation analysis was conducted using the Pearson’s *r*. All values in the figures represent mean ± SEM. All statistical analyses were performed with GraphPad Prism software, version 9 (GraphPad Software, Inc), and comparisons were considered statistically significant if *p* < 0.05.

## Data availability

All data presented in this article are available upon request.

## Supporting information

This article contains supporting information (68).

## Conflict of interest

J. G.-M. had a sponsored research contract with *NeuRistic*. All the other authors declare that they have no conflicts of interest with the contents of this article.

## Supporting information

Supplementary tables

FigureS1
