# Supplementary material for: Glucocorticoid receptor dysregulation underlies 5-HT2AR-dependent synaptic and behavioral deficits in a mouse neurodevelopmental disorder model
Source: J Biol Chem. 2022 Sep 12;298(11):102481. doi: 10.1016/j.jbc.2022.102481 (PMC9589215; doi:10.1016/j.jbc.2022.102481)
Supplement: Supplementary tables [file mmc1.pdf]

Supplementary Tables:

Table S1. Three-way ANOVA analysis of the effect of MIA on %PPI in male and female C57BL6/N CRL mice.

Source of variation	ANOVA	p value
Prepulse intensity	F[2,147] = 46.82	p < 0.001
MIA	F[1,147] = 12.01	p < 0.001
Sex	F[1,147] = 3.42	p > 0.05
Prepulse intensity × MIA	F[2,147] = 0.03	p > 0.05
Prepulse intensity × Sex	F[2,147] = 0.009	p > 0.05
MIA × Sex	F[1,147] = 0.98	p > 0.05
Prepulse intensity × MIA × Sex	F[2,147] = 0.25	p > 0.05

Table S2. Three-way ANOVA analysis of the effect of MIA on frontal cortex dendritic spine density (stubby) in male and female 129S6/SvEv in-house mice

Source of variation	ANOVA	p value
Genotype	F[1,359] = 7.97	p < 0.01
MIA	F[1,359] = 4.46	p < 0.05
Sex	F[1,359] = 1.66	p > 0.05
Genotype × MIA	F[1,359] = 0.01	p > 0.05
Genotype × Sex	F[1,359] = 6.43	p < 0.05
MIA × Sex	F[1,359] = 1.62	p > 0.05
Genotype × MIA × Sex	F[1,359] = 0.14	p > 0.05

Table S3. Three-way ANOVA analysis of the effect of MIA on frontal cortex dendritic spine density (thin) in male and female 129S6/SvEv in-house mice

Source of variation	ANOVA	p value
Genotype	F[1,359] = 0.03	p > 0.05
MIA	F[1,359] = 4.80	p < 0.05
Sex	F[1,359] = 123.3	p < 0.001
Genotype × MIA	F[1,359] = 1.69	p > 0.05
Genotype × Sex	F[1,359] = 0.23	p < 0.05
MIA × Sex	F[1,359] = 3.29	p > 0.05
Genotype × MIA × Sex	F[1,359] = 1.65	p > 0.05

Table S4. Three-way ANOVA analysis of the effect of MIA on frontal cortex dendritic spine density (mushroom) in male and female 129S6/SvEv in-house mice

Source of variation	ANOVA	p value
Genotype	F[1,359] = 12.24	p < 0.001
MIA	F[1,359] = 14.42	p < 0.001
Sex	F[1,359] = 0.03	p > 0.05
Genotype × MIA	F[1,359] = 7.89	p < 0.01
Genotype × Sex	F[1,359] = 11.34	p < 0.001
MIA × Sex	F[1,359] = 11.46	p < 0.001
Genotype × MIA × Sex	F[1,359] = 2.94	p > 0.05

Table S5. Three-way ANOVA analysis of the effect of MIA on frontal cortex dendritic spine density (total) in male and female 129S6/SvEv in-house mice

Source of variation	ANOVA	p value
Genotype	F[1,359] = 4.63	p < 0.05
MIA	F[1,359] = 10.29	p < 0.01
Sex	F[1,359] = 27.02	p < 0.001
Genotype × MIA	F[1,359] = 0.08	p > 0.05
Genotype × Sex	F[1,359] = 5.50	p < 0.05
MIA × Sex	F[1,359] = 6.51	p < 0.05
Genotype × MIA × Sex	F[1,359] = 0.002	p > 0.05

Table S6. List of primer pairs

	Primer Set		Sequence
**Genotyping Primers**			
	5-HT$_{2A}$R Genotyping	Wild Type Fwd	GTGTGATGGCTCTTGATTATGC
		5-HT$_{2A}$R KO Fwd	TCTCTTGATTCCCACTTTGTGGTT
		Common Rev	CTGTGGGATTTTCTTTCTGCTT
**Microbiome Primers**			
	SFB	SFB736	GACGCTGAGGCATGAGAGCAT
		SFB884	GACGGCACGGATTGTTATTCA
	Universal Bacteria	UniF340	ACTCCTACGGGAGGCAGCAGT
		UniR514	ATTACCGCGGCTGCTGGC
**qPCR Primers**			
	IL-6	Fwd	TAGTCCTTCCTACCCCAATTTCC
		Rev	TTGGTCCTTAGCCACTCCTTC
	5-HT$_{2A}$R	Fwd	GCAGTCCATCAGCAATGAGC
		Rev	GCAGTGGCTTTCTGTTCTCC
	5-HT$_{2C}$R	Fwd	GTATTCCCTCCCTTCCTTGC
		Rev	CGTGTGTGAATGAGCAGAGC
	D_2	Fwd	CTCTACCCTCCAATCCACTC
		Rev	CATCCACAGCCTCCTCTAAG
	GR	Fwd	TTCGCAGGCCGCTCAGTGTT
		Rev	TTGGGAGGTGGTCCCGTTGCT
	FKBP4	Fwd	GAGGAAATGCAAAAGGTCCA
		Rev	CTTCTCGTTGTTGCTGTCCA
	FKBP5	Fwd	GTACAACAAAGCCGTGGAGTG
		Rev	GCCCTGTTCTGAGGATTGACT
	Actin	Fwd	AGAGGGAAATCGTGCGTGAC
		Rev	CAATAGTGATGACCTGGCCGT
	GAPDH	Fwd	TGCGACTTCAACAGCAACTC
		Rev	CTTGCTCAGTGTCCTTGCTG
	MAPK5	Fwd	CATTGCCCAGTGTATCCTCC
		Rev	ACCTGCTTTACCACCTCTGC
	rpS3	Fwd	AGGTTGTGGTGTCTGGGAAG
		Rev	GAGGCTTCTTGGGACCAATC
**ChIP Primers**			
	-361 to -204	Fwd	CCATCCCTCCTGGACACATCA
		Rev	GTCATATTTTTAGGCTGAGGGGTG
	+92 to +201	Fwd	GATTCCTCTCTGTGCGCTCG
		Rev	TTCCAGCACGGTTGAAGTCT
	+698 to +886	Fwd	AGCAGCATATCCAACCCGAGAA
		Rev	TGGCTCTTGATTATGCCTCGC
	+1003 to +1137	Fwd	GACTCGCTAGTCTCTCCACA
		Rev	GCCTCGAGTCGTCACCTAAT
	+1170 to +1340	Fwd	TCCGAAGCCTCGAACTGGA
		Rev	CCAGTATGTTTCCCGCAATGG
	+1692 to +1880	Fwd	CCTGTATTCCAATACTCTGTGAGC
		Rev	AGCACTAATGCTCTCTCTGCAA
**Cloning Primers**			
	ΔGR Cloning	Fwd	TTTTGGATCCATGGACTCCAAAGAATCCTT
		Rev	TTTTGGCGCGCCTGAGACTCCTGCAGTGGCTT
**FISH Probe Primers**			
	5-HT$_{2A}$R Antisense Probe	Fwd	GCAGAATGCCACCAACTATTTC
		Rev	TTTACCTGGATGTGCTCTTCTC
	Negative Control Sense Probe	Fwd	GAGAAGAGCACATCCAGGTAAA
		Rev	GAAATAGTTGGTGGCATTCTGC
