# Supplementary material for: Glucocorticoid receptor dysregulation underlies 5-HT2AR-dependent synaptic and behavioral deficits in a mouse neurodevelopmental disorder model
Source: J Biol Chem. 2022 Sep 12;298(11):102481. doi: 10.1016/j.jbc.2022.102481 (PMC9589215; doi:10.1016/j.jbc.2022.102481)
Supplement: FigureS1 [file mmc2.pdf]

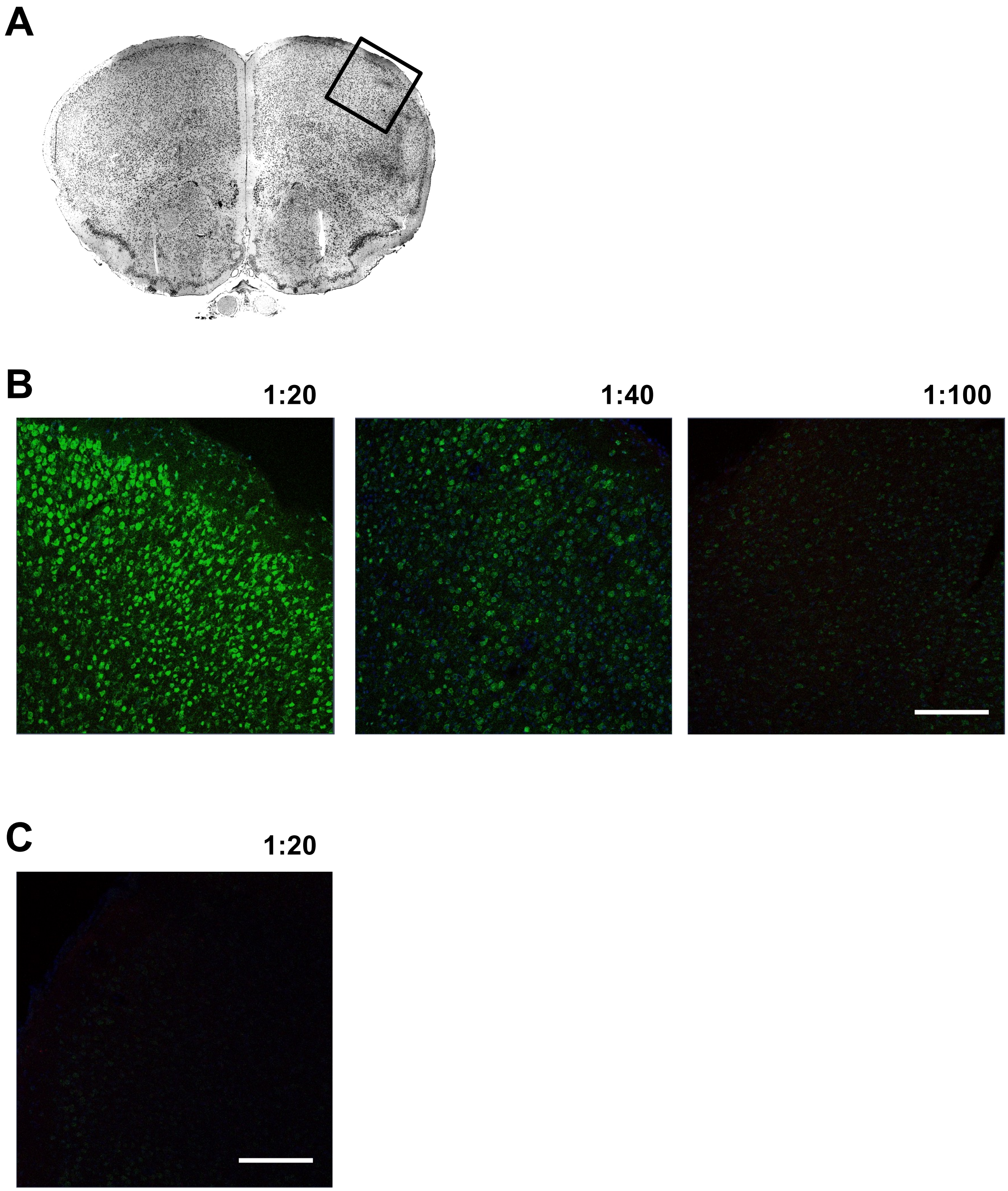

Figure S1. Control assays validate the FISH protocol with the antisense *5-HT$_{2A}$R* probe in mouse frontal cortex. *A,* Nissl-staining image of mouse frontal cortex for neuroanatomical orientation. Image was taken from the mouse brain atlas with author's permission (68). Region of interest is marked by a box. *B*, FISH signal in female C57BL6/N CRL mouse frontal cortex decreases with increasing dilution factors of the antisense *5-HT$_{2A}$R* probe. *C,* Signal is absent with a sense *5-HT$_{2A}$R* probe. Scale bars, 200 µm (*B,C*).
